# Inhibition of NUPR1–Karyopherin β1 Binding Increases Anticancer Drug Sensitivity

**DOI:** 10.3390/ijms22062794

**Published:** 2021-03-10

**Authors:** Chanhee Park, Jiwon Oh, Won Mo Lee, Hye Ran Koh, Uy Dong Sohn, Seung Wook Ham, Kyungsoo Oh

**Affiliations:** 1 Center for Metareceptome Research, College of Pharmacy, Chung-Ang University, 84 Heukseok-ro, Dongjak, Seoul 06974, Korea; parkch09@yuhs.ac (C.P.); lwm6313@naver.com (W.M.L.); udsohn@cau.ac.kr (U.D.S.); 2 Institute of Gastroenterology, College of Medicine, Yonsei University, 50-1 Yonsei-ro, Seodaemun, Seoul 03772, Korea; 3 Department of Chemistry, Chung-Ang University, 84 Heukseok-ro, Dongjak, Seoul 06974, Korea; ohjiwon16@gmail.com (J.O.); hrkoh@cau.ac.kr (H.R.K.)

**Keywords:** KPNB1, NUPR1, protein binding, drug sensitivity, combination

## Abstract

Background: Nuclear protein-1 (NUPR1, also known as p8/Com-1) is a transcription factor involved in the regulation of cellular stress responses, including serum starvation and drug stimulation. Methods: We investigated the mechanism of NUPR1 nuclear translocation involving karyopherin β1 (KPNB1), using a single-molecule binding assay and confocal microscopy. The cellular effects associated with NUPR1–KPNB1 inhibition were investigated by gene expression profiling and cell cycle analysis. Results: The single-molecule binding assay revealed that KPNB1 bound to NUPR1 with a binding affinity of 0.75 nM and that this binding was blocked by the aminothiazole ATZ-502. Following doxorubicin-only treatment, NUPR1 was translocated to the nucleus in more than 90% and NUPR1 translocation was blocked by the ATZ-502 combination treatment in MDA-MB-231 with no change in NUPR1 expression, providing strong evidence that NUPR1 nuclear translocation was directly inhibited by the ATZ-502 treatment. Inhibition of KPNB1 and NUPR1 binding was associated with a synergistic anticancer effect (up to 19.6-fold) in various cancer cell lines. NUPR1-related genes were also downregulated following the doxorubicin–ATZ-502 combination treatment. Conclusion: Our current findings clearly demonstrate that NUPR1 translocation into the nucleus requires karyopherin β1 binding. Inhibition of the KPNB1 and NUPR1 interaction may constitute a new cancer therapeutic approach that can increase the drug efficacy while reducing the side effects.

## 1. Introduction

The transcription factor nuclear protein-1 (NUPR1, aka p8/Com-1) is a member of the high-mobility group of transcriptional regulators [1], and it plays an important role in several different biological processes, including transcription, cell cycle, autophagy, and cell death [2,3,4]. NUPR1 is also a component of the cellular stress response, playing a role in serum starvation, oxidative stress, and drug stimulation, and it is overexpressed in various cancers [5,6,7]. NUPR1 expression is known to be upregulated after anticancer drug treatment (doxorubicin, gemcitabine, paclitaxel, celecoxib, and sorafenib) in breast and liver cancer cells [8,9], resulting in the downstream activation of the PI3K/AKT signaling pathway and CDKN1A/p21 phosphorylation [8,10,11]. NUPR1 is also involved in the translocation of p27 from the nucleus to cytoplasm in pancreatic cells. The elucidation of the translocation mechanism of NUPR1 is expected to provide important information concerning the resistance of cancer drugs and their extrinsic cytotoxicity [12].

Karyopherin β1 (KPNB1, known as importin β1) is a carrier protein functioning as a transporter of proteins from the cytoplasm to the nucleus through nuclear pore complexes, using KPNAs as adapters [13,14]. In addition, KPNB1 is involved in mitosis-related cellular mechanisms, including mitotic spindle assembly, microtubule–kinetochore attachment, mitotic exit, and nuclear envelop assembly [14,15,16,17]. Previously, we reported that (*E*)-N-(5-benzylthiazol-2-yl)-3-(furan-2-yl) acrylamide (aminothiazole, ATZ-502) showed cytotoxic effects in several cancer cells by blocking the function of KPNB1 with pSTAT3, STAT3, EGFR, and ErbB2 [18,19,20,21].

Doxorubicin-containing adjuvant chemotherapy was recommended as a first-line anticancer drug in the 2016 NCCN breast cancer guidelines, especially for patients with HER-2 positive and triple-negative breast cancer [22]. To lower the doxorubicin dose, drug combination treatments with other cancer drugs, including docetaxel and trastuzumab, have been examined; however, while no significant therapeutic indication has been observed, more severe cardiotoxicity side effects have been observed at lower cumulative doses [23]. Thus, research efforts to overcome the cytotoxicity of this important cancer drug have not been fruitful, mainly because the biochemical mechanisms of doxorubicin are not well understood [24].

Given the multiple functional roles of NUPR1 in anticancer drug treatment, we hypothesized that the downstream effects of NUPR1 target binding do not require a change in NUPR1 expression levels. The downstream effects of NUPR1 may occur through direct binding or indirect binding to one or multiple protein targets. In the current study, we found that NUPR1 binds to the nuclear import receptor karyopherin β1, thus translocating from the cytoplasm to the nucleus. Our findings should help in contributing to a further understanding of the biochemical mechanisms of extrinsic cytotoxicity and cancer drug resistance. Moreover, it is hoped that our results may provide evidence that the regulation of NUPR1 and karyopherin β1 binding may provide a new approach for cancer therapy through drug combination treatment.

## 2. Results

### 2.1. Dose- and Time-Dependent Cytotoxic Effects of Doxorubicin on MDA-MB 231 Breast Cancer Cells

**Figure 1:**
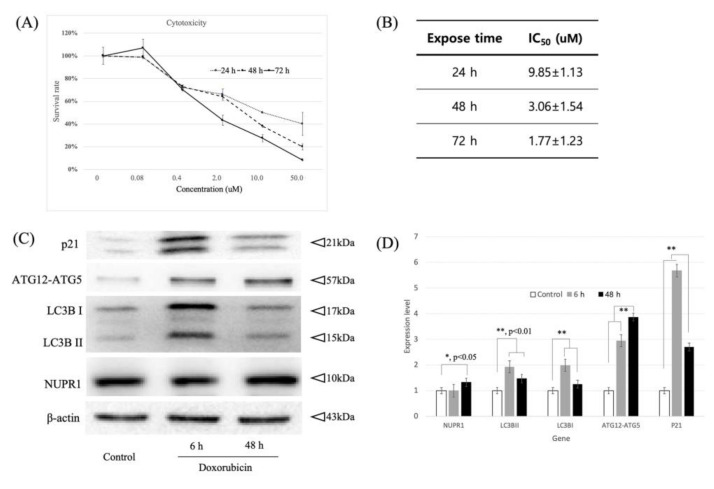
Effect of doxorubicin exposure time on cytotoxicity and NUPR1-related protein expression in MDA-MB-231 breast cancer cells. (**A**) Increasing concentrations of doxorubicin were assayed in MDA-MB-231 cells using a time-dependent approach. (**B**) Table indicating the IC_50_ values of doxorubicin, calculated for MDA-MB-231 breast cancer cells. Cells were seeded in 48-well plates and incubated with increasing concentrations of doxorubicin. Values represent mean±SD of at least three individual experiments. MDA-MB-231 breast cancer cells were treated with 1 μM of doxorubicin for 6 and 48 h. Immunoblot images (**C**) and quantification (**D**) for NUPR1, p21, and autophagy-related protein (ATG12-ATG5, LC3B1, and LC3BII) levels as assessed by Western blot. β-Actin was used as loading control. *, *p* < 0.05; **, *p* < 0.01; bars, SD.

MDA-MB 231 breast cancer cells readily develop doxorubicin resistance following doxorubicin treatment [25,26]. MDA-MB 231 cells were treated with doxorubicin for 24, 48, and 72 h to investigate the effects of doxorubicin cytotoxicity. The IC_50_ of doxorubicin against MDA-MB 231 cells was subsequently calculated using CalcuSyn software. Doxorubicin inhibited MDA-MB-231 cell viability in a dose- and time-dependent manner from 24 to 72 h (Figure 1A). The IC_50_ values of doxorubicin on MDA-MB-231 cells for 24- and 72-h exposure were 9.85 and 1.77 μM, respectively (Figure 1B). The apoptosis-related p21 gene and autophagy-related genes for LC3BI, LC3BII, and ATG12-ATG5 were induced by the doxorubicin treatment. While NUPR1 protein levels did not change in the first 6 h of doxorubicin treatment, NUPR1 protein levels increased significantly by 48 h (Figure 1C,D).

### 2.2. Increased NUPR1 Nuclear Transportation after Doxorubicin Treatment on MDA-MB-231 Breast Cancer Cells

**Figure 2:**
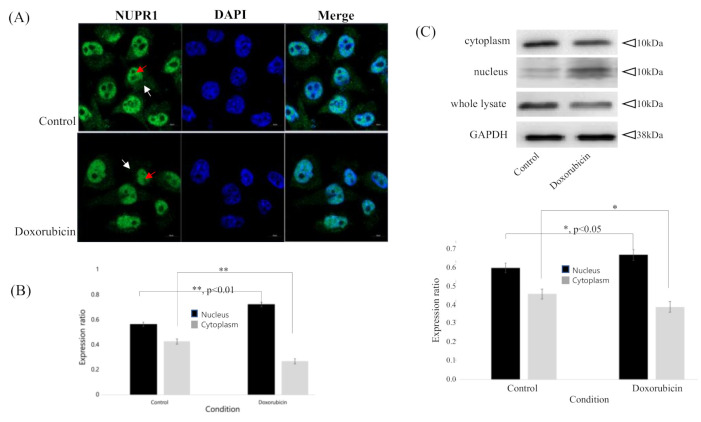
Cellular localization of NUPR1 protein after treatment with doxorubicin in MDA-MB231 cells. MDA-MB-231 breast cancer cells were treated with doxorubicin for 4 h. (**A**,**B**) NUPR1 protein was located in both cytoplasm (43.1%) and nucleus (56.9%) without doxorubicin. NUPR1 localization in the nucleus (72.9%) was increased after doxorubicin treatment (1 μM) for 4 h, compared to cytoplasm (27.1%). Cellular localization of NUPR1 was observed under a confocal microscope. (**C**) NUPR1 protein expression in nucleus was increased following doxorubicin treatment according to Western blot analysis (compared to control). Images were obtained using a Carl Zeiss LSM 800 confocal microscope and visualized to assess protein localization. White arrow, cytoplasm; Red arrow, nucleus; *, *p* < 0.05; **, *p* < 0.01; bars, SD.

According to our results, doxorubicin treatment induces an increase in autophagy-related protein and p21 protein expression before any changes in NUPR1 expression are observed (Figure 1C,D). To confirm the change in subcellular localization of NUPR1 protein, an immunofluorescence assay was performed on MDA-MB-231 cells at 4 h after treatment with 1 μM doxorubicin (Figure 2A). In the doxorubicin-only treatment group, NUPR1 was largely localized within the nuclei (72.9%) of MDA-MB-231 cells (Figure 2B), while a small proportion remained in the cytoplasm (27.1%). In comparison, NUPR1 localization of the control group was higher in the cytoplasm (43.1%) and lower in the nucleus (56.9%) (Figure 2A,B). The change in NUPR1 protein distribution was confirmed by Western blot analysis of nuclear and cytoplasmic fractions (Figure 2C). While NUPR1 protein in the nucleus was increased at 4 h after the doxorubicin-only treatment (compared to the control group), NUPR1 expression in whole cell lysate was unchanged. These results provide strong evidence for the translocation of NUPR1 from the cytoplasm to the nucleus within a 6-h period after doxorubicin treatment.

### 2.3. ATZ-502 Inhibits NUPR1 Activity in MDA-MB-231 Breast Cancer Cells by Inhibiting Nuclear Transportation

**Figure 3:**
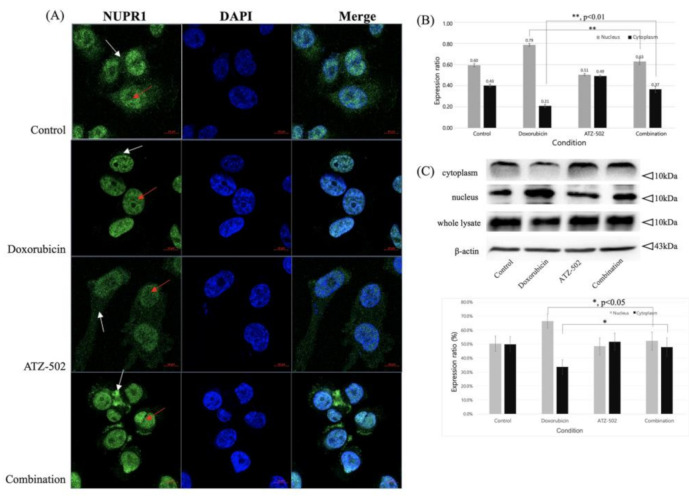
Changes in NUPR1 cellular localization after combination treatment to MDA-MB231 cells. The breast cancer cell line MDA-MB-231 was treated with doxorubicin only and doxorubicin plus ATZ-502 combination for 4 h. (**A**,**B**) NUPR1 protein was located within both cytoplasm and nucleus. The doxorubicin and ATZ-502 single-treated cells were grown in the presence of 1 μM doxorubicin and 1 μM ATZ-502, respectively. The combination-treated cells were grown in the presence of 1 μM each of doxorubicin and ATZ-502 for 2 h. Cellular localization of NUPR1 was observed under a confocal microscope. Images were obtained using a ZEISS LSM 800 confocal microscope and visualized to assess protein localization. (**C**) NUPR1 and karyopherin β1 protein expression levels in nucleus and cytoplasm using Western blot analysis. White arrow, cytoplasm; Red arrow, nucleus. *, *p* < 0.05; **, *p* < 0.01; bars, SD.

To further investigate the observed changes in NUPR1 protein subcellular localization in MDA-MB-231 cells, an immunofluorescence assay was performed at 2 h after the treatment with doxorubicin only (1 μM), aminothiazole (ATZ-502) only (1 μM), and doxorubicin (1 μM) plus ATZ-502 (1 μM), (Figure 3A,B). In the doxorubicin-only treatment group, NUPR1 was localized in the nucleus in more than 90% of the MDA-MB-231 cells. In the combination-treatment group, NUPR1 was localized in both the nucleus and the cytoplasm in more than 90% of the MDA-MB-231 cells, indicating that NUPR1 translocation to the nucleus was inhibited by the ATZ-502 treatment. The results are consistent with the proposal that ATZ-502 blocks the transport of NUPR1 into the nucleus by inhibiting NUPR1 binding to KPNB1.

### 2.4. Inhibition of NUPR1 Nucleus Transportation Induces G2/M Phase Arrest and Increases Synergistic Cytotoxicity Effects

**Figure 4:**
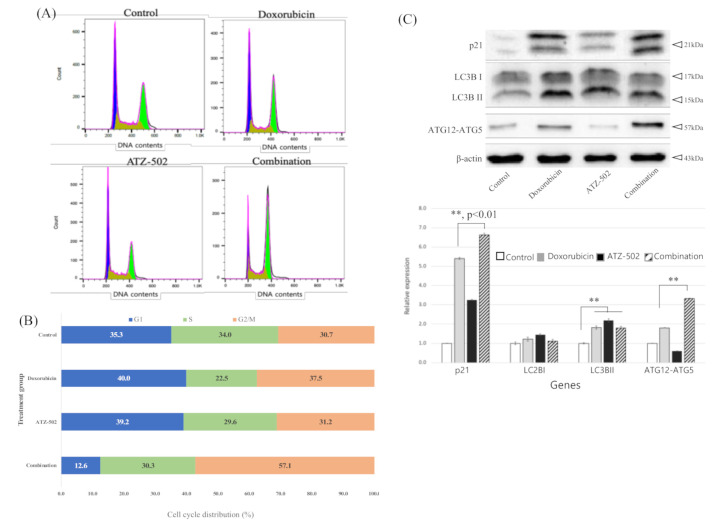
Cell cycle analysis of MDA-MB 231 cells. (**A**) Cell cycle analysis of MDA-MB 231 cells by FACS after treatment with doxorubicin only (1 μM), ATZ-501 (1 μM) only, and the combination treatment of both doxorubicin (1 μM) and ATZ-502 (1 μM). (**B**) Representative histogram graph of the cell cycle analysis of MDA-MB 231 cells. After combination treatment, MDA-MB 231 cells were inhibited in the G2/M phase (in comparison to the single treatments). (**C**) Change in autophagy-related proteins (ATG12-ATG5, LC3B1, and LC3BII) after combination treatment with doxorubicin and ATZ-502. **, *p* < 0.01; bars, SD.

The effects of inhibition of NUPR1 nuclear transportation on cell cycle distribution in MDA-MB 231 cells were analyzed using fluorescence-activated cell sorting (FACS) analysis. The results revealed that 40.0% and 12.6% of cells were in the G0/G1 phase following the doxorubicin-only and the combination treatment, respectively, and 37.5% and 57.1% of cells were in the G2/M phase following the doxorubicin-only and the combination treatment, respectively (*p* < 0.05) (Figure 4A,B). No significant difference was observed between the control and the ATZ-502-only treatment groups. These data indicated that the cell cycle was arrested in the G2/M phase following the doxorubicin and ATZ-502 combination treatment. Additionally, p21 protein and autophagy-related protein expression levels were increased, as demonstrated by Western blot analysis (Figure 4C). Our results suggest that ATZ-502 treatment inhibits the doxorubicin-induced NUPR1 nuclear transportation with an associated increase in p21/ATG12-ATG5/LC3BI/LC3BII and G2/M phase arrest.

To verify that the effects of combination treatment were not a cell-type-specific artifact, the cytotoxicity assay was performed in several different cancer cell lines: a breast cancer cell line (MDA-MB-231); an ovarian cancer cell line (SK-OV-3); a cervical cancer cell line (SiHa); a prostate cancer cell line (PC-3); and a doxorubicin-resistant breast cancer cell line (MCF-7/ADR). The IC_50_ values for the single and combination treatments were determined in the above cell lines using the Ez-Cytox assay (Supplementary Figure S1A–E). To decouple the cytotoxic effect of ATZ-502 from the doxorubicin and ATZ-502 combination effect, the cytotoxic effects of ATZ-502 were independently verified in MDA-MB 231 cells. After the treatment with different concentrations of ATZ-502, the IC_50_ of ATZ-502 was calculated to be >4 μM (Supplementary Figure S1F). During our investigations of the effect of doxorubicin and ATZ-502 combination treatment on the different cell lines, the concentration of ATZ-502 was varied between 0.01 and 0.5 μM to minimize cellular damage in the individual cell lines. To further investigate cellular changes after combination treatment, we performed the FACS analysis. After combination treatment for 24 h, the numbers of early apoptotic cells were slightly elevated compared to the single treatment (Supplementary Figure S1G–J).

The cytotoxic effects of combination treatment against MDA-MB-231, PC-3, MCF-7/ADR, SK-OV-3, and SiHa are shown in Table 1. The results indicate that the inhibition of NUPR1 nuclear transportation following the doxorubicin and ATZ-502 combination treatment is accompanied by synergistic cytotoxic effects. In particular, the doxorubicin sensitivity was increased 19.6-fold in MCF-7/ADR and PC-3 cells; thus, the IC_50_ values of doxorubicin in these two cell lines were changed from 39.62 and 0.357 μM to 2.0 and 0.018 μM, respectively (Table 1). The other cell lines also demonstrated a similar (but less dramatic) synergistic cytotoxic effect: MDA-MB-231, 1.767 to 0.438 μM (4-fold); SiHa, 0.624 to 0.239 μM (2.6-fold); and SK-OV-3, 0.250 to 0.175 μM (1.4-fold) (Table 1).

### 2.5. RNA Quant-Seq Analysis Revealed That Single and Combination Treatments Yielded Different mRNA Expression Patterns

**Figure 5:**
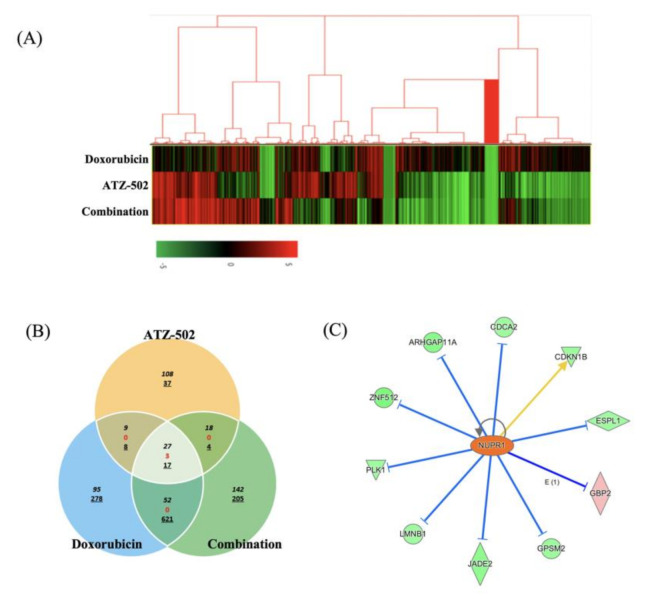
Differentially expressed gene (DEG) selection and identification of regulated genes using gene expression profiling. (**A**) Supervised hierarchical clustering of up- and downregulated genes between the control and the treated cells (2-fold) (Red, upregulated; Green, downregulated. (**B**) Selection of common DEGs between the single- and the combination-treatment profiles (Black, commonly overexpressed genes; Red, change in gene expression patterns between compared groups; Underline, commonly downregulated genes). (**C**) After Ingenuity Pathway Analysis (IPA) analysis, 10 NUPR1-regulated genes were selected from 347 DEGs. Of these genes, nine genes were downregulated and one gene was upregulated more than 2-fold compared to the control. (Yellow and blue line, prediction of relation).

RNA Quant-seq analysis was performed to investigate changes in gene expression. The differentially expressed gene (DEG) sets include all genes with a >2-fold difference between the control and the treatment group. In the MDA-MB-231 cell line, 1624 genes were differentially expressed in the treatment groups (compared with the control group). The overlap set containing DEGs from the single- and the combination-treatment groups included 27 upregulated genes and 17 downregulated genes. The DEG set for the doxorubicin–ATZ-502 combination-treatment group (Figure 5A) comprised 142 upregulated genes and 205 downregulated genes (Supplementary Table S1). These included 47 common DEGs (shared by the single- and the combination-treatment groups): 27 upregulated genes; 17 downregulated genes; and 3 genes showing the opposite expression pattern (Figure 5B).

### 2.6. Gene Ontology Analysis for DEGs

A core analysis was performed on the single-treatment group and combination-treatment group DEG sets using Ingenuity Pathway Analysis (IPA) on specific canonical pathways. In the doxorubicin-only treatment group, genes in the prolactin signaling pathway, the allergic inflammatory airway disease pathway by IL-17F, the Wnt/Ca^2+^ pathway, and the osteoarthritis pathway demonstrated significant gene expression changes. In the ATZ-502-only treatment group, genes in the estrogen receptor signaling pathway, the osteoarthritis pathway, the H1F1a signaling pathway, and the pigment epithelium-derived factor signaling pathway were markedly activated. In the combination-treatment group, genes in the inositol pyrophophate biosynthesis pathway, the 1D-myo-inositol hexakisphosphate biosynthesis V pathway, the dermatan sulfate biosynthesis pathway, the notch signaling pathway, and the dermatan sulfate biosynthesis pathway were differentially expressed (Supplementary Table S2). For a preliminary verification of differential expression, seven genes from the overlap set containing DEGs from the single- and combination-treatment groups were chosen for further analysis (Figure 5B). The mRNA expression levels for these selected genes were validated using qRT-PCR, and the results were compared with the corresponding protein expression levels from Western blot analyses of MDA-MB-231 cells (Supplementary Figure S2A–C).

### 2.7. NUPR1-Induced Gene Expression Is Differentially Regulated between Single-Treatment and Combination-Treatment Groups

The change in the expression pattern of NUPR1-related genes was investigated for the doxorubicin-only treatment and the doxorubicin–ATZ-502 combination treatment. The 347 DEGs related to cancer and gastrointestinal disease in the combination treatment were first examined. From within this gene set, the following genes were identified as NUPR1-downregulated genes: Rho GTPase activating protein 11A (*ARHGAP11A*); cell division cycle associated 2 (*CDCA2*); extra spindle pole bodies like 1 (*ESPL1*); jade family PHD finger 2 (*JADE2*); lamin B1 (*LMNB1*); polo-like kinase 1 (*PLK1*); and zinc finger protein 512 (*ZNF512*). Thus, mRNA expression of these genes was downregulated (more than 2-fold) in the combination group (compared with the control group). Likewise, the guanylate binding protein 2 (*GBP2*) and the G protein signaling modulator 2 (*GPSM2*) genes were upregulated more than 2-fold (Figure 5C). To validate mRNA and protein expression, the *NUPR1* and *PLK1* genes were selected. In the NUPR1 case, while no significant differences in protein expression were observed, mRNA expression was slightly upregulated (less than 2-fold) in the doxorubicin-only and combination-treatment groups, and downregulated (more than 2-fold) in the ATZ-502-treatment group (Supplementary Figure S2D,E). In contrast, *PLK1* protein expression was increased in the doxorubicin-only treatment group, although no difference in *PLK1* mRNA expression was observed. Interestingly, *PLK1* mRNA and *PLK1* protein expression levels both decreased in the combination-treatment group.

### 2.8. Karyopherin β1 Binds to NUPR1 with High Affinity

**Figure 6:**
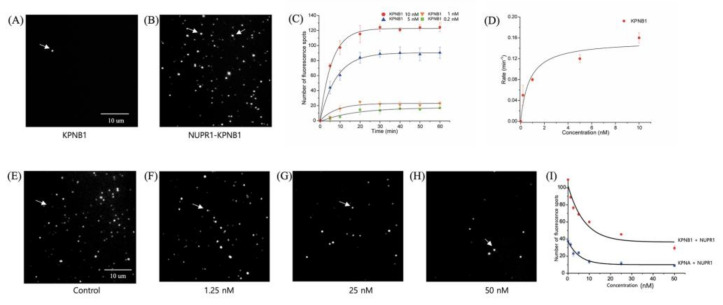
Single-molecule binding assay. (**A**) Karyopherin β1 (5 nM) was rarely bound to the imaging surface in the absence of immobilized NUPR1 protein. (**B**) Significant increase in karyopherin β1 (5 nM) binding to the NUPR1-immobilized quartz surface. (**C**) Number of NUPR1-binding spots over time at various concentrations of karyopherin β1. (**D**) The apparent dissociation rate constants obtained at different concentration of karyopherin β1, yielding a Km of 0.75 nM after fitting to a Michaelis–Menten equation. (**E**–**H**) Single-molecule fluorescence images showing karyopherin β1 binding to the NUPR1-immobilized imaging surface in the presence of various concentrations of ATZ-502: (**E**) 0; (**F**) 1.25; (**G**) 25; and (**H**) 50 nM. (**I**) Karyopherin β1 and karyopherin α binding to the NUPR1-immobilized imaging surface was monitored in the presence of various concentrations of ATZ-502. Karyopherin β1 and karyopherin α were preincubated with ATZ-502 for 30 min on ice. White arrow, NUPR1–karyopherin β1 binding spots.

Proteins are transported into the nucleus by forming a complex with the nuclear transport proteins karyopherin α/karyopherin β1 (encoded by the *KPNA*, *KPNB1* genes, respectively) in the classical transport pathway, or with karyopherin β1 alone in the non-classical transport pathway [18]. To examine the binding mode of NUPR1, a single-molecule pull-down assay was employed that enabled the monitoring of protein–protein interaction at the single-molecule level in real time (Supplementary Figure S3A). Each binding event involving NUPR1 and karyopherin β1 was visualized as a single fluorescent spot on the imaging surface (Figure 6A,B). To obtain the binding kinetics for the NUPR1 and karyopherin β1 interaction, the number of binding spots was monitored at various time points after the addition of karyopherin β1 to NUPR1 (Figure 6C and Figure S4A). The observed rate constant (*k_obs_*), reflecting the association and dissociation of NUPR1–karyopherin β1 complex, was estimated from an exponential fitting at 5 nM karyopherin β1. This method yielded a *k_obs_* of 0.12 min^−1^. Likewise, the dissociation rate constant (*k_off_*) for the NUPR1–karyopherin β1 complex was estimated from an exponential fitting of the dissociation data, which was obtained by counting the number of remaining bound spots at specified time points after washing out all the unbound karyopherin β1 in the imaging solution (Supplementary Figure S4B). This method yielded a *k_off_* of 0.018 min^−1^. The on-rate constant (*k_on_*) for the binding interaction between NUPR1 and karyopherin β1 was calculated as 0.028 min^−1^ nM^−1^, using *k_obs_* = *k_on_*[karyopherin β1] − *k_off_*. Therefore, the corresponding dissociation constant (*K_m_* = *k_off_*/*k_on_*) for the NUPR1–karyopherin β1 complex was calculated to be 0.65 nM (approx.), demonstrating the high-affinity binding interaction between karyopherin β1 and NUPR1. To further confirm the dissociation constant, we measured the observed rate constants at various different concentrations of karyopherin β1 (Figure 6C) and then fitted these values using a Michaelis–Menten equation, yielding a dissociation constant of 0.75 nM (Figure 6D). This was consistent with the results calculated from the on-rate constant and the off-rate constant obtained at 5 nM karyopherin β1, supporting the strong binding affinity between karyopherin β1 and NUPR1.

### 2.9. ATZ-502 Inhibits the NUPR1–Karyopherin β1 Interaction

Compound ATZ-502 inhibits nuclear transportation by binding to karyopherin β1 [18,19,20,21]. In the current study, ATZ-502 inhibition was demonstrated in a quantitative manner by directly monitoring the number of karyopherin β1 and NUPR1 binding events after the addition of various concentrations of ATZ-502 (Supplementary Figure S3B) (Figure 6E–H). The ATZ-502 inhibition of NUPR1–karyopherin β1 binding was clearly demonstrated from the decreased number of karyopherin β1 and NUPR1 binding events at high ATZ-502 concentrations. These results provide further support to the finding that NUPR1 strongly binds to karyopherin β1 (Figure 6A–D and Figure S4C), not karyopherin α (Figure 6I), suggesting the non-classical transport pathway.

## 3. Discussion

In the present study, we established the importance of the protein–protein binding interaction between NUPR1 and karyopherin β1 in the protein nuclear translocation mechanism, and we demonstrated that ATZ-502 inhibited this interaction.

The NUPR1 protein, known as an early stress response-related gene, has anti-apoptotic and anti-inflammatory functions, and NUPR1-deficient mice present increased apoptosis [27]. NUPR1 also inhibits protein synthesis and regulates cell proliferation and autophagy, thus modulating cellular stress-associated cell death [5]. The current experimental results are in agreement with the results of several previous reports and confirm that the NUPR1 expression was increased during 48 h after doxorubicin treatment. However, according to our results, NUPR1 protein was rapidly relocated from the cytoplasm into the nucleus within 4 h of the doxorubicin treatment, without a change in protein level (Figure 2A–C). While NUPR1 protein was translocated to the nucleus in more than 90% of cells after the doxorubicin-only treatment, the NUPR1 translocation was markedly blocked by ATZ-502 (Figure 3A–C). Although there was no significant change in the NUPR1 protein expression immediately following doxorubicin treatment, the expression levels of NUPR1-regulated genes, including autophagy-related genes (Figure 1C,D) and other NUPR1-regulated genes (Figure 5C), were changed. These results strongly support the hypothesis that the NUPR1 protein expression does not change during the early cellular stress response to doxorubicin treatment. However, NUPR1 protein does undergo translocation from the cytoplasm into the nucleus during the early stages of the cellular stress response. Thus, the current results validate our hypothesis concerning the involvement of NUPR1 in direct protein target binding without any apparent change in NUPR1 expression levels.

The nuclear import receptor karyopherin β1 (encoded by *KPNB1*) is involved in drug resistance in various cancer cells, including B-cell lymphoma [28], glioblastoma [29], ovarian, and esophageal carcinoma cells [30]. Previously, the aminothiazole compound ATZ-502 was identified as an effective inhibitor of karyopherin β1 function, showing a binding affinity of ~20 nM (*Kd*) [18,19]. The cytotoxic effect of ATZ-502 was demonstrated in several cancer cells by blocking the nuclear translocation of pSTAT3, STAT3, EGFR, and ErbB2 by karyopherin β1 [18,19,20,21]. Thus, it is reasonable to speculate about the importance of a high-affinity protein–protein interaction between karyopherin β1 and NUPR1. As described in Figure 6, the ATZ-502 effectively inhibited the protein–protein binding between KPNB1 and NUPR1 in a dose-dependent manner. Furthermore, the doxorubicin and ATZ-502 combination treatment clearly demonstrates a synergistic cytotoxic effect in various cancer cells, including MDA-MB-231, SiHa, PC-3, and SK-OV-3 cells, of up to 19.6-fold (Table 1). Additionally, to validate the synergistic effect of NUPR1–karyopherin β1 binding inhibition, we performed a new combination treatment using Importazole (IPZ, a known karyopherin β1 inhibitor). Again, combination treatment with doxorubicin and IPZ showed a synergistic effect (Supplementary Figure S5A,B).

According to our analysis of gene expression profiling, there was no significant difference in the NUPR1 mRNA expression levels; however, NUPR1-regulated genes were downregulated in the doxorubicin and ATZ-502 combination treatment group compared to the doxorubicin-only treatment group (Figure 5). Of these NUPR1-regulated genes, polo-like kinase 1 (*PLK1*) is known to be involved in the p53-related pathway, and the depletion of PLK1 induced apoptosis [31]. Another NUPR1-regulated gene, the cell cycle-associated 2 gene (*CDCA2*) is typically overexpressed in tumor cells and is positively correlated with tumor proliferation [32].

In summary, the observed synergistic cytotoxic effects of doxorubicin–ATZ-502 combination treatment in various cancer cell lines support the hypothesis that nuclear translocation of NUPR1 requires the NUPR1 binding to karyopherin β1. Thus, inhibition of the protein–protein interaction between NUPR1 and karyopherin β1 by small molecules is a candidate drug combination strategy that may be broadly applicable in cancer therapeutics.

## 4. Materials and Methods

### 4.1. Cell Culture

MDA-MB-231 and MCF-7/ADR (Breast), PC-3 (Prostate), SiHa (Cervix), SK-OV-3 (Ovarian) cancer cell lines were obtained from the American Type Culture Collection (ATCC, Manassas, VA, USA). MDA-MB-231, PC-3, and SK-OV-3 cells were cultured in RPMI-1640 (WELGENE, Daegu, Korea), and MCF-7/ADR and SiHa cells were cultured in DMEM (WELGENE, Daegu, Korea) media with 10% fetal bovine serum (Corning, VA, USA) and 1% penicillin–streptomycin and then maintained in incubator at 37 °C with 5% CO_2_ humidified air. The experiments were performed in accordance with the guidelines of the Institutional Animal Care Use Committee of Chung-Ang University (Seoul, Korea).

### 4.2. Cell Viability Assay

The cytotoxic effects of doxorubicin (Sigma-Aldrich, Munich, Germany) and aminothiazole (ATZ-502) were analyzed by using EZ-Cytox kit (DoGenBio, Seoul, Korea). In brief, MDA-MB-231 and PC-3 cells were seeded at 4 × 10^4^ cells/well in a 24-well plate for 24 h and then added with each reagent. After 24, 48, and 72 h incubation, 10 μL of EZ-Cytox was treated to each well and incubated for 1 h at 37 °C. Absorbance was monitored at 450 nm with a microplate reader (Molecular Devices, CA, USA). Cell viability was decided by comparison with the vehicle-treated control samples. The combination index (CI) was calculated using the Chou–Talalay method [33]. The combination index (CI) assesses the synergistic effect (CI < 1), additive effect (CI = 1), or antagonistic effect (CI > 1).

### 4.3. Cell Cycle and Apoptosis with Fluorescence-Activated Cell Sorting (FACS) Analysis

The change in cell cycle was analyzed by using FACS analysis after the doxorubicin (Sigma-Aldrich, Munich, Germany) and aminothiazole (ATZ-502) treatment. After the MDA-MB 231 cells were seeded in 6-well plates for 24 h, the cells were treated with the single and the combination treatment for 24 h. After the 24-h treatment, the cells were collected from the culture plates using 0.25% trypsin and rinsed with cold PBS. Subsequently, the cells were fixed with ice-cold 70% ethanol and incubated at −20 °C for 18 h. The cells were subsequently resuspended in cold PBS and incubated with 1 mL propidium iodide (PI) staining solution (containing 50 μg/mL propidium iodide and 50 μg/mL RNase A) at ambient temperature for 25 min in the dark. For the analysis of apoptosis, the washed cells were labeled with Muse Annexin V and dead cell assay kit (Merck Millipore, Darmstadt, Germany) for 30 min. The detection of the labeled cells was performed using a Muse Cell Analyzer (Merck Millipore, Germany). The distribution of cells in each cell cycle phase was evaluated by flow cytometry (BD FACSCalibur, CA, USA) using CellQuest Pro software (BD Biosciences, CA, USA).

### 4.4. Single-Molecule Binding Assay

The protein–protein interaction at the single-molecule level was investigated by using a home-built total internal reflection fluorescence (TIRF) microscopy. Details of the surface passivation and optical configurations were the same as previously described [34]. Briefly, to measure the binding affinity of NUPR1 to KPNB1 protein, NUPR1 recombinant protein (NOVUS Biologicals, CO, USA) was immobilized on the PEG-coated quartz surface by adding 20 μL of 0.05 mg/mL neutravidin (Thermo Fisher Scientific, DE, USA), 100 μL of 10 nM biotin-conjugated GST-tag antibody (Abcam, MA, USA), and 100 μL of 100-nM GST-NUPR1 sequentially. For each addition, it was incubated for 5–10 min. Then, 50 μL of 5-nM Cy5-labeled KPNB1 in the imaging buffer (4 mM Trolox, 0.2% (*w*/*v*) glucose, 50 mM NaCl, pyranose oxidase (3.1 mg/100 μL), 2170 U/mL catalase) was added to the NUPR1-immobilized imaging surface. Each binding between NUPR1 to KPNB1 protein was visualized as a single fluorescence spot by a TIRF microscope. The number of fluorescence spots was counted up to 1 h after the addition of KPNB1 to NUPR1-immobilized imaging surface to measure the binding kinetics of KPNB1 to NUPR1 (Supplementary Figure S3A). After 1 h, the KPNB1 in the imaging solution was washed out, and the number of remaining fluorescence spots that showed the binding between KPNB1 and NUPR1 was counted to measure the dissociation rate of NUPR1 from KPNB1.

### 4.5. Drug Inhibition Assay at the Single-Molecule Level

To test the inhibition effect of ATZ-502 on the binding between NUPR1 and KPNB1, the NUPR1 was immobilized on the imaging surface and then 5 nM of Cy5-labeled KPNB1 or Cy5-labeled KPNA was added. The resulting mixture was incubated with various concentrations of ATZ-502 for 30 min. The extent of Cy5-KPNB1 binding to NUPR1 in the presence of ATZ-502 was visualized with excitation with a 633-nm laser at ambient temperature (Supplementary Figure S3B).

### 4.6. Immunofluorescence Assay

Cells were seeded in a confocal dish (SPL Life Sciences, Gyeonggi-do, Korea) and then cultured in single (doxorubicin, or ATZ-502) and combination (doxorubicin and ATZ-502) treatments for 4 h. Immunofluorescent staining of cells was performed as previously described [35,36]. Briefly, confocal dishes were fixed with the fix buffer consisting of 4% formaldehyde (Cell Signaling Technology, MA, USA) for 15 min at room temperature. After washing three times with wash buffer (Cell Signaling Technology, MA, USA), cells were then permeabilized with 100% cold methanol for 10 min at −20 °C and treated with blocking buffer (Cell Signaling Technology, MA, USA) for 1 h at ambient temperature. Primary antibody, anti-NUPR1 (NOVUS Biologicals, CO, USA, 1:200), for immunofluorescence was incubated overnight at 4 °C. After washing three times, cells were incubated with the secondary antibody Alexa Fluor 488 (Abcam, MA, USA, 1:1000) for 1 h under dark conditions. Each confocal dish was observed on a ZEISS LSM 800 confocal microscope (ZEISS, Jena, Germany). Single and 3D images were assessed in a ZEN system of ZEISS image software (ZEISS, Jena, Germany).

### 4.7. Gene Expression Profiling Analysis Using QuantSeq 3′ mRNA-seq

For mRNA expression profiling, the quality of extracted total RNA was analyzed by an Agilent 2100 bioanalyzer (Agilent Technologies, Amstelveen, The Netherlands) and ND-2000 Spectrophotometer (Thermo Fisher Scientific, DE, USA). The QuantSeq 3′ mRNA-seq was performed as per the manufacturer’s instructions [37]. Briefly, the construction of a library was performed using the QuantSeq 3′ mRNA-Seq Library Prep Kit (Lexogen, Vienna, Austria) with 500 ng total RNA. After the construction of the library, high-throughput sequencing was performed as single-end 75 sequencing using NextSeq 500 (Illumina, CA, USA).

### 4.8. Data Analysis and Gene Selection

QuantSeq 3′ mRNA-Seq reads were aligned using Bowtie2 [38]. Bowtie2 indices were either generated from the genome assembly sequence or the representative transcript sequences for aligning to the genome and transcriptome. The alignment file was used for assembling transcripts, estimating their abundances, and detecting the differential expression of genes.

Differentially expressed genes were selected more than 2-fold differences between the control and the treated samples. The RT (Read Count) data were processed based on the global normalization method using the Genowiz version 4.0.5.6 (Ocimum Biosolutions, Hyderabad, India). Hierarchical clustering analysis was performed with Cluster software, in which the clustering algorithm was used with a complete linkage and Pearson correlation, and the resulting dendrogram was visualized using TreeView software (http://diyhpl.us/~bryan/irc/protocol-online/protocol-cache/EisenSoftware.htm; Accessed on 8 March 2021). The biological function and molecular mechanical relationships of the selected genes were investigated using Core Analysis in Ingenuity Pathways Analysis (IPA) 5.5 (Ingenuity Systems, CA, USA). The annotation of selected genes was performed using the Database for Annotation, Visualization, and Integrated Discovery (DAVID; https://david.ncifcrf.gov; Accessed on 8 March 2021) and the Stanford Online Universal Resource for Clones and Expressed sequence tags (SOURCE; https://source-search.princeton.edu; Accessed on 8 March 2021). For the optimal biological validation, the probes targeting intronic sequences were excluded and the values of multiple exonic probes for the same gene were averaged.

### 4.9. Quantitative Real-Time RT-PCR

Cells were grown to confluence and the total RNA was separated using a TRIzol reagent kit (Invitrogen, CA, USA) according to the manufacturer’s instructions. RNA was reverse-transcribed using Cycle script (Bioneer, Daejoen, Korea) and an oligo (DT) primer. The sequences of the primers used in this study were Actin, forward primer 5′-CGA GCT GTC TTC CCA TCC A-3′ and reverse primer 5′-TCA CCA ACG TAG CTG TCT TTC TG-3′; MNT, forward 5′-TCC TTC CCT TGT TGT GTG TG-3′, reverse 5′-CAC GGG GAG AAA AAT CAA TG-3′; SOCS1, forward 5′-GCA GAC CCC TTC TCA CCT CT-3′, reverse 5′-CCC TGG TTT GTG CAA AGA TA-3′; TNS4, forward 5′-GGC CTG ACG GTT ATG ATT TC-3′, reverse 5′-GAG TAT ATG GGG GCC TCT GC-3′; CYBA, forward 5′-GAG CGG CAT CTA CCT ACT GG-3′, reverse 5′-GGT TGA CCT GGG GAC CTC-3′; LGMN, forward 5′-GCT CCA GGA CCT TCT TCA CA-3′, reverse 5′-ATG AGC TTC CTG CTC CTC AA-3′; HSP90B1, forward 5′-TGG ATG TGG GAA CAG ATG AA-3′, reverse 5′-GGG CAT CCA AAA CAA GTC TC-3′; HDAC7, forward 5′-TCC CAC CCC ACA TAG GAG TA-3′, reverse 5′-ACA GGG TAT GGT GGG TCC TA-3′. Data were calculated using Bio-Rad CFX Manager Software.

### 4.10. Protein Expression, Immunoprecipitaton, and Western Blotting

For the comparison of protein expression, the total protein was extracted using NP-40 lysis buffer (20 mM Tris/HCl, pH 8.0, 150 mM NaCl, 1% Nonidet P40, 10% (*v*/*v*) glycerol, 2.5 mM EDTA, 100 mM Na_3_VO_4_, 1% aprotinin, 1% leupeptin, and 1 mM phenylmethanesulfonyl fluoride). The nuclear protein was extracted using the NE-PER Nuclear and Cytoplasmic Extraction Kit (Thermo Fisher Scientific, DE, USA), according to the manufacturer’s instructions. For the validation of protein–protein interaction, the immunoprecipitation (IP) analysis was performed. First, 50 µL of protein G magnetic beads (Millipore, MA, USA) and the KPNB1 antibody (described in Western blot methods above) were gently mixed at ambient temperature for 30 min. After washing, the immobilized capture antibody (described in Western blot methods above) was bound with the KPNB1–NUPR1 protein complex and this binding was inhibited by ATZ-502 (1 µM) at 4 °C overnight. RIPA buffer was added for denaturation and elution. The extracted and immunoprecipitated protein were separated by sodium dodecyl sulfate (SDS) polyacrylamide gel electrophoresis using 12% polyacrylamide gels. Proteins were transferred onto polyvinyl difluoride membranes (GE Healthcare, WI, USA) and analyzed by immunoblotting using antibodies against NUPR1, 1:500 (NOVUS Biologicals, CO, USA), PLK1 (Santa Cruz Biotechnology, TX, USA), KPNB1 (Cell signaling, MA, USA), LC3B (Cell signaling, Danvers, MA, USA), Atg12-Atg5 (Cell Signaling Technology, MA, USA), and p21 (Cell Signaling Technology, MA, USA), β-actin, 1:2000 (Santa Cruz Biotechnology, CA, USA). Secondary anti-mouse immunoglobulin G (IgG)-horseradish peroxidase (HRP) (GE Healthcare, WI, USA) and anti-goat IgG-HRP (Santa Cruz Biotechnology, CA, USA) were used at 1:2000 and 1:1000 dilutions, respectively. The membrane was developed with ECL Western blotting reagents (Amersham Pharmacia Biotech, WI, USA), and the signal intensity was analyzed using ImageJ image analysis software (NIH).

### 4.11. Statistical Analysis

The cell viability assay (triplicate experiments independently repeated at least two times) and each molecular experiment were performed more than 3 times and the quantification of confocal images was counted from more than 5 cells. Each datum was averaged and statistically analyzed by Student’s *t*-test. To investigate the synergistic efficacy of the drug combination, the combination index (CI) was determined according to the Chou–Talalay method using CalcuSyn software version 2.1 (Biosoft, Cambridge, UK) [33]. A p-value less than 0.05 was considered significant.

## Figures and Tables

**Table 1:**
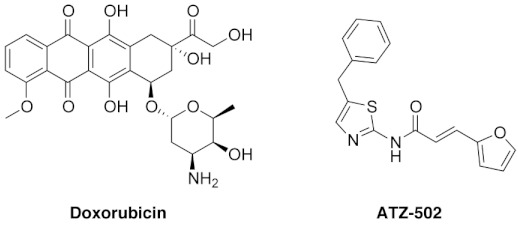
IC_50_ of single and combination treatment with doxorubicin and ATZ-502. ATZ-502 concentration was determined with IC_50_ after cytotoxicity assay. Cell lines were treated with a range of doxorubicin concentrations as indicated to assess the combination effect with ATZ-502. Cell lines were exposed to drugs for 72 h. The Ez-Cytox reagent (20 μL) was administered to each well, and the absorbance measurements were taken at 450 nm. All values are averages of replicates expressed relative to the cell viability values in the untreated cells normalized to 100%. Cytotoxicity represents four replicates per drug concentration for each experiment.

Cell Lines	ATZ-50 (μM)	IC_50_ (μM)		Fold Difference
		Single	Combination	
MDA-MB-231	0.50	1.767	0.438	4.0
SiHa	0.50	0.624	0.239	2.6
MCF-7/ADR	0.05	39.62	2.00	19.8
SK-OV-3	0.01	0.250	0.175	1.4
PC-3	0.01	0.357	0.018	19.6

## Data Availability

We included all data in supplementary information and additional data that support the finding of this study are available from the corresponding author upon reasonable request.

## Supplementary Materials

The following are available online at https://www.mdpi.com/1422-0067/22/6/2794/s1, Figure S1: Cell lines were treated with a range of doxorubicin concentrations as indicated to assess the combination effect with ATZ-502, S2: Validation of mRNA and protein expression, S3: Illustration of single-molecule binding assay, S4: KPNB1 and NUPR1 binding assay Table S1: RNA Quant-seq analysis of doxorubicin-ATZ-502 combination treatment, S2: Top 5 Ingenuity Cannonical Pathway, S3: Combination Index (CI) values for doxorubicin and ATZ-502 combination with various cancer cell lines.
