# Supplementary material for: Inhibition of NUPR1–Karyopherin β1 Binding Increases Anticancer Drug Sensitivity
Source: Int J Mol Sci. 2021 Mar 10;22(6):2794. doi: 10.3390/ijms22062794 (PMC8000408; doi:10.3390/ijms22062794)
Supplement: Supplementary file 1 [file ijms-22-02794-s001.zip › Supplementary Materials /Supplementary Figures.pptx]

## Slide 1
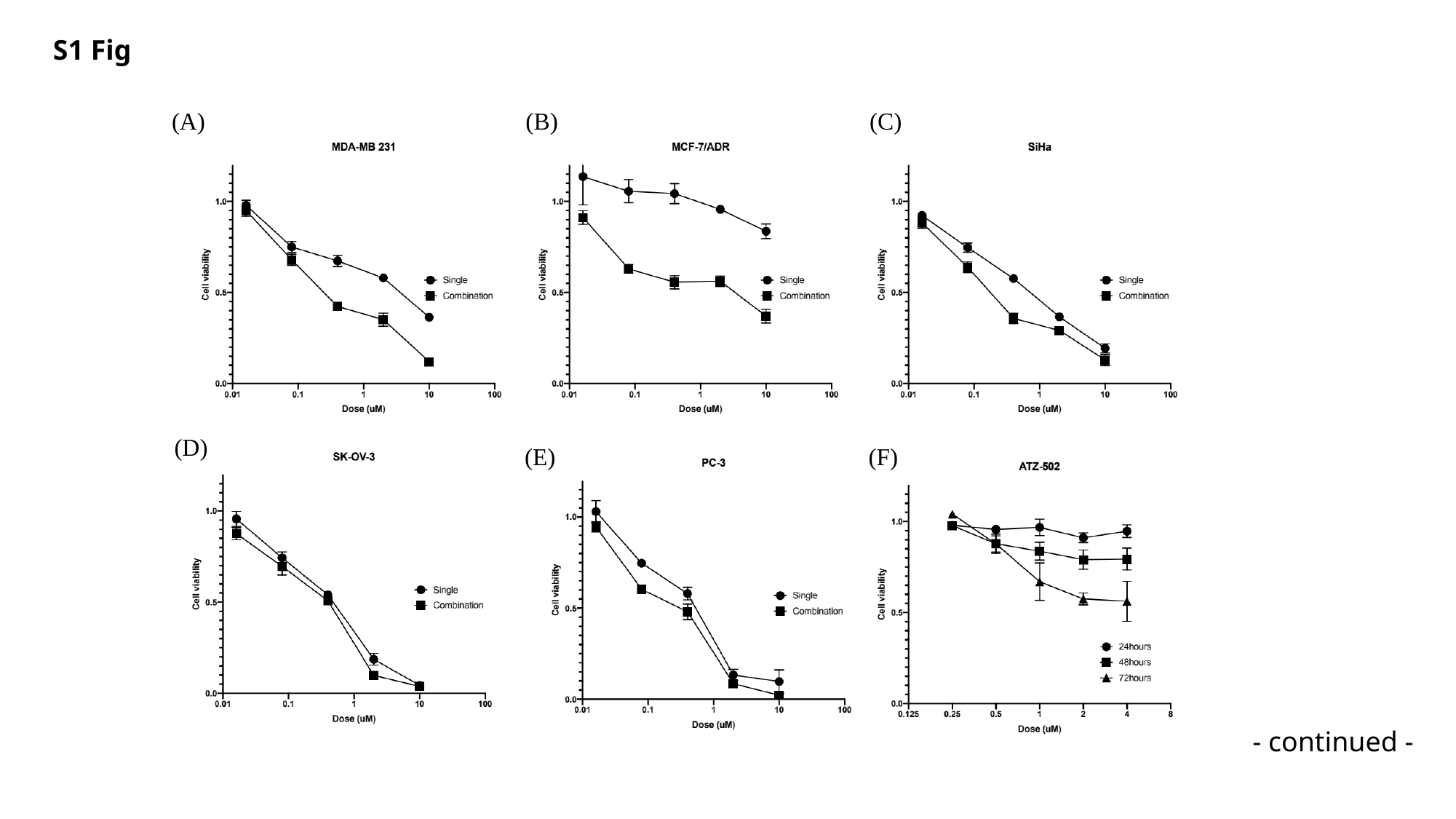

S1 Fig
(C)
(B)
(A)
(D)
(E)
(F)
 - continued -

## Slide 2
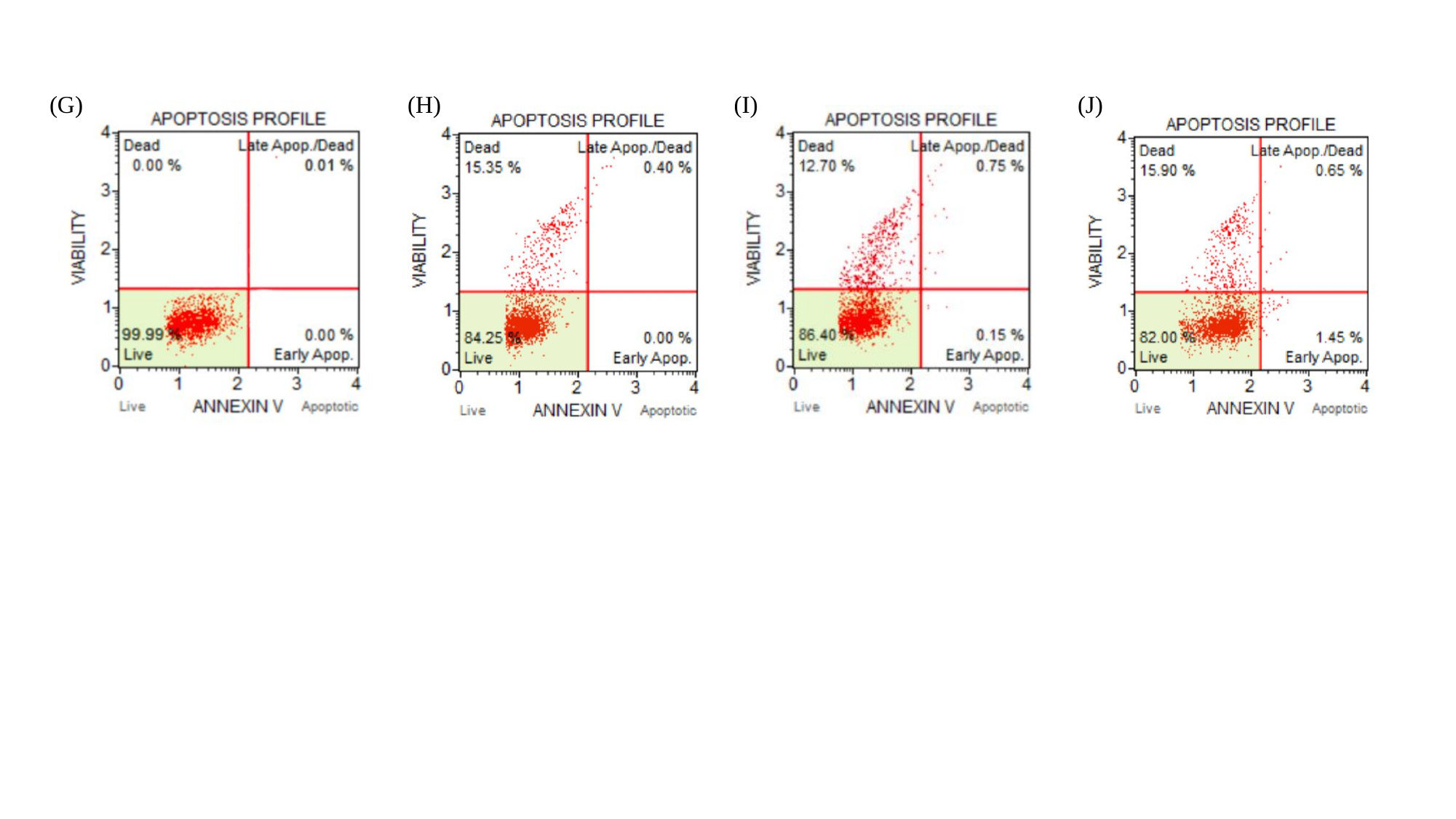

(J)
(G)
(H)
(I)

## Slide 3
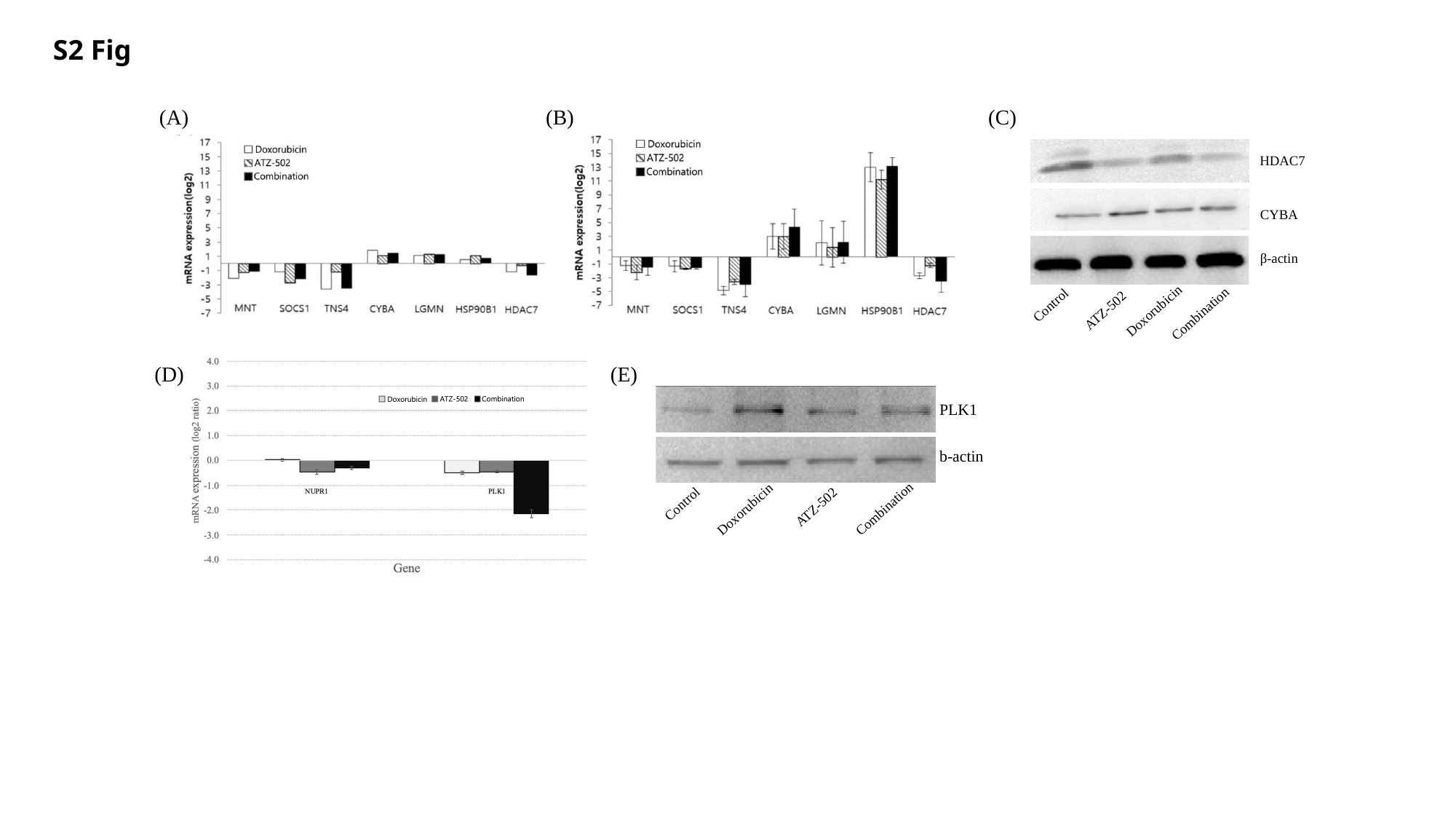

S2 Fig
(C)
(A)
(B)
HDAC7
CYBA
β-actin
Control
ATZ-502
Doxorubicin
Combination
(E)
(D)
PLK1
b-actin
Control
ATZ-502
Combination
Doxorubicin

## Slide 4
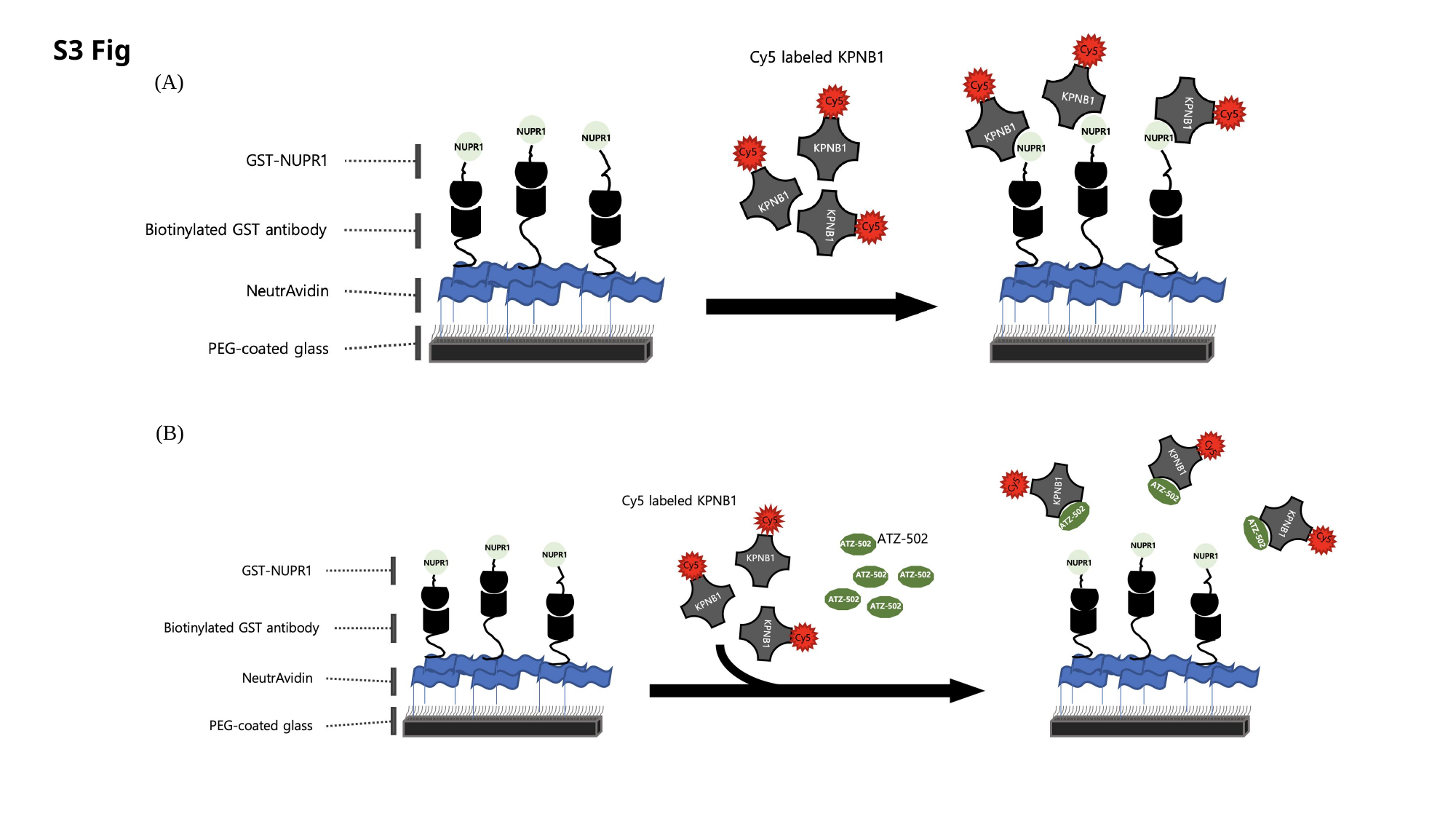

S3 Fig
(A)
(B)

## Slide 5
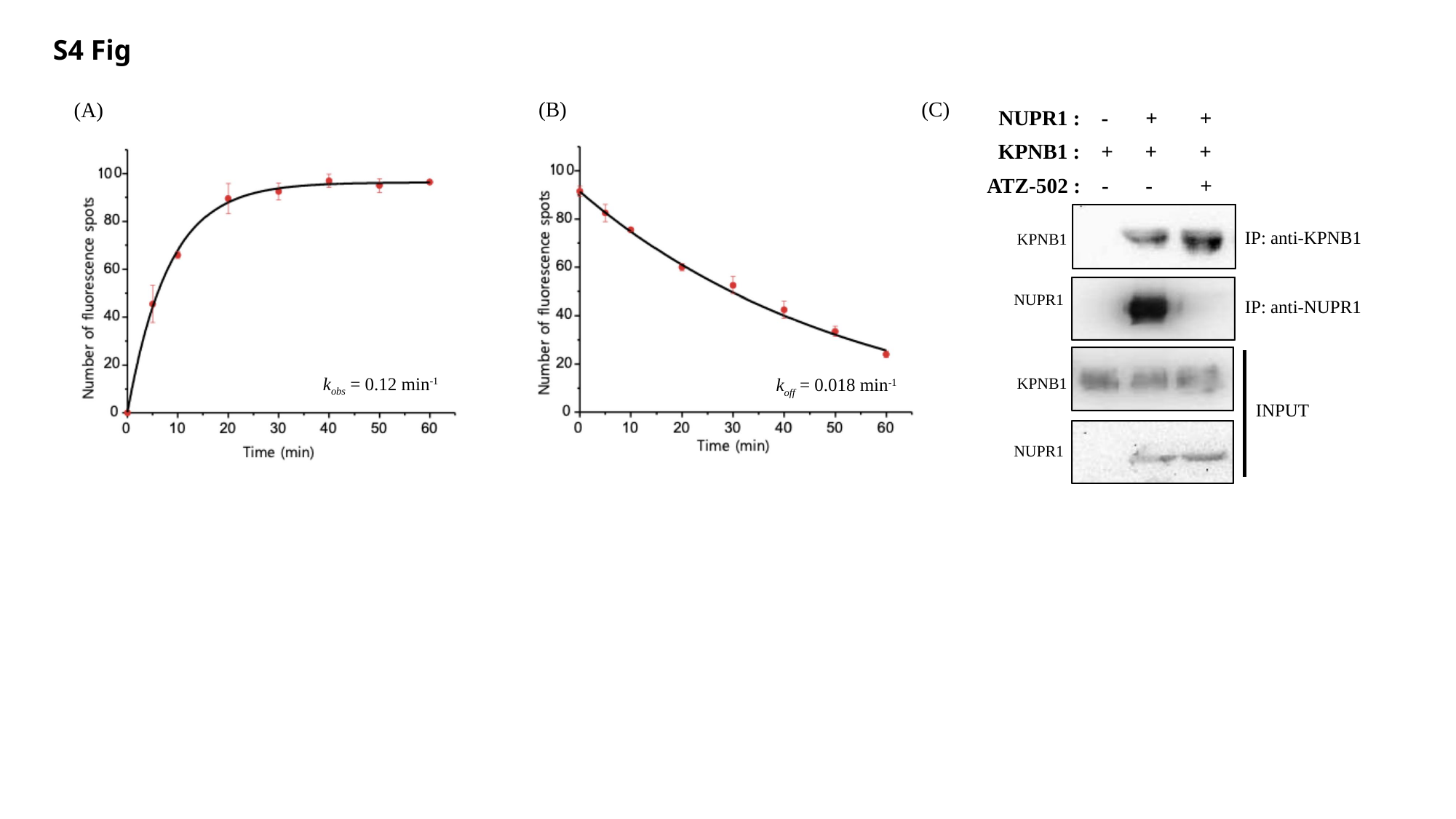

S4 Fig
(B)
(C)
(A)
NUPR1 : - + +
KPNB1 : + + +
ATZ-502 : - - +
IP: anti-KPNB1
KPNB1
NUPR1
IP: anti-NUPR1
kobs = 0.12 min-1
koff = 0.018 min-1
KPNB1
INPUT
NUPR1

## Slide 6
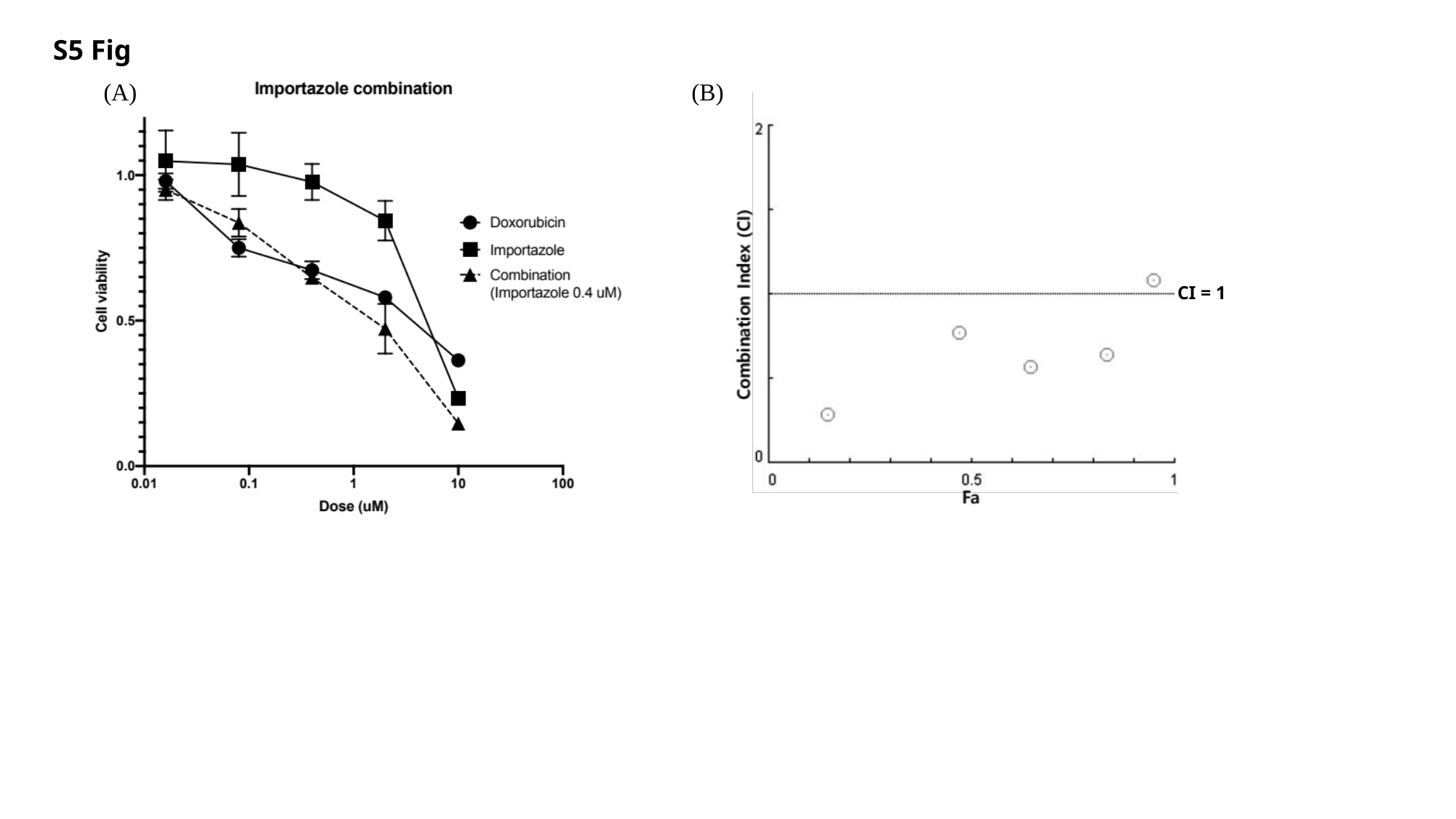

S5 Fig
(A)
(B)
CI = 1
