# Supplementary material for: Inhibition of NUPR1–Karyopherin β1 Binding Increases Anticancer Drug Sensitivity
Source: Int J Mol Sci. 2021 Mar 10;22(6):2794. doi: 10.3390/ijms22062794 (PMC8000408; doi:10.3390/ijms22062794)
Supplement: Supplementary file 1 [file ijms-22-02794-s001.zip › Supplementary Materials /Supplementary materials.docx]

**Inhibition of NUPR1-Karyopherin β1 Binding Increases Anticancer Drug Sensitivity**

Chanhee Park,^1,2^ Jiwon Oh,^3^ Won Mo Lee,^1^ Hye Ran Koh,^3^ Uy Dong Sohn,^1^ Seung Wook Ham,^3,*^ Kyungsoo Oh^1,*^

^1^Center for Metareceptome Research, College of Pharmacy, Chung-Ang University, 84 Heukseok-ro, Dongjak, Seoul 06974, Republic of Korea

^2^Institute of Gastroenterology, College of Medicine, Yonsei University, 50-1 Yonsei-ro, Seodaemun, Seoul 03772, Republic of Korea

^3^Department of Chemistry, Chung-Ang University, 84 Heukseok-ro, Dongjak, Seoul 06974, Republic of Korea

*corresponding authors: [swham@cau.ac.kr](mailto:swham@cau.ac.kr); [kyungsoooh@cau.ac.kr](mailto:kyungsoooh@cau.ac.kr)

**
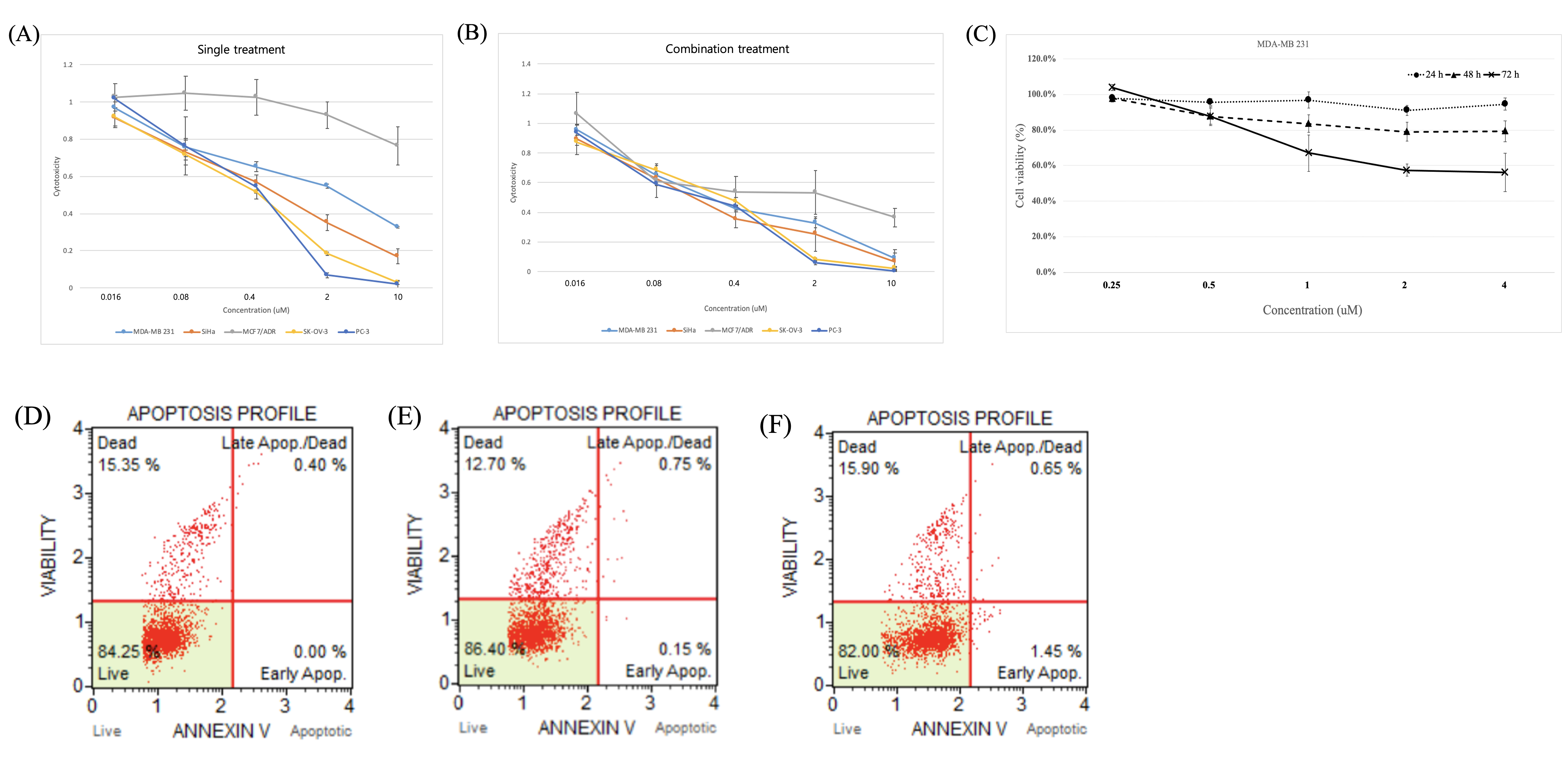
**

**Supplementary Figure 1.** Cell lines were treated with (A) a range of doxorubicin concentrations as indicated to assess (B) the combination effect with ATZ-502. Cell lines were exposed to compounds for 72 h. 20 μl of Ez-CyTox reagent was administered to each well, and the absorbance measurements were taken at 450 nm. All values are averages of replicates expressed relative to cell viability values in untreated cells normalized to 100%. Cytotoxicity represents 4 replicates per compound concentration for each experiment. Flow cytometric analysis of MDA-MB 231 cells following (D) Doxorubicin, (E) ATZ-502 and (F) Combination.

**
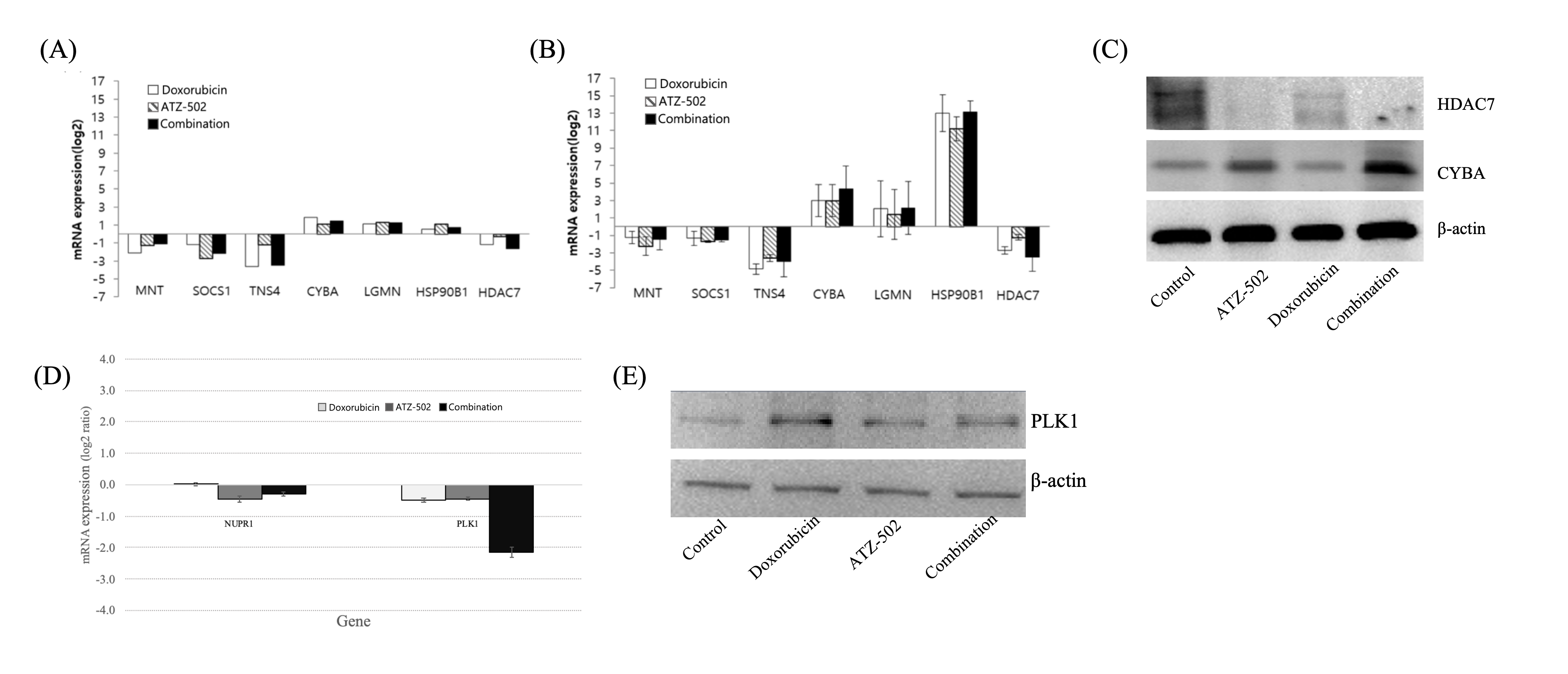
**

**Supplementary Figure 2**. Validation of mRNA and protein expression. (A) Relative mRNA expression was analyzed by gene expression profiling. (B) Total RNA was isolated using TRIzol reagent and relative mRNA quantity was calculated by quantitative real-time PCR (qPCR). Values indicated mean±SD of at least 3 individual experiments. In these selected genes, western blot analysis was performed with HDAC7 and CYBA (C). Selected 10 NUPR1 related DEGs, mRNA and protein expression of PLK1 gene was validated using qRT-PCR (D) and western blot (E) in MDA-MB-231 after 4 h treatment.


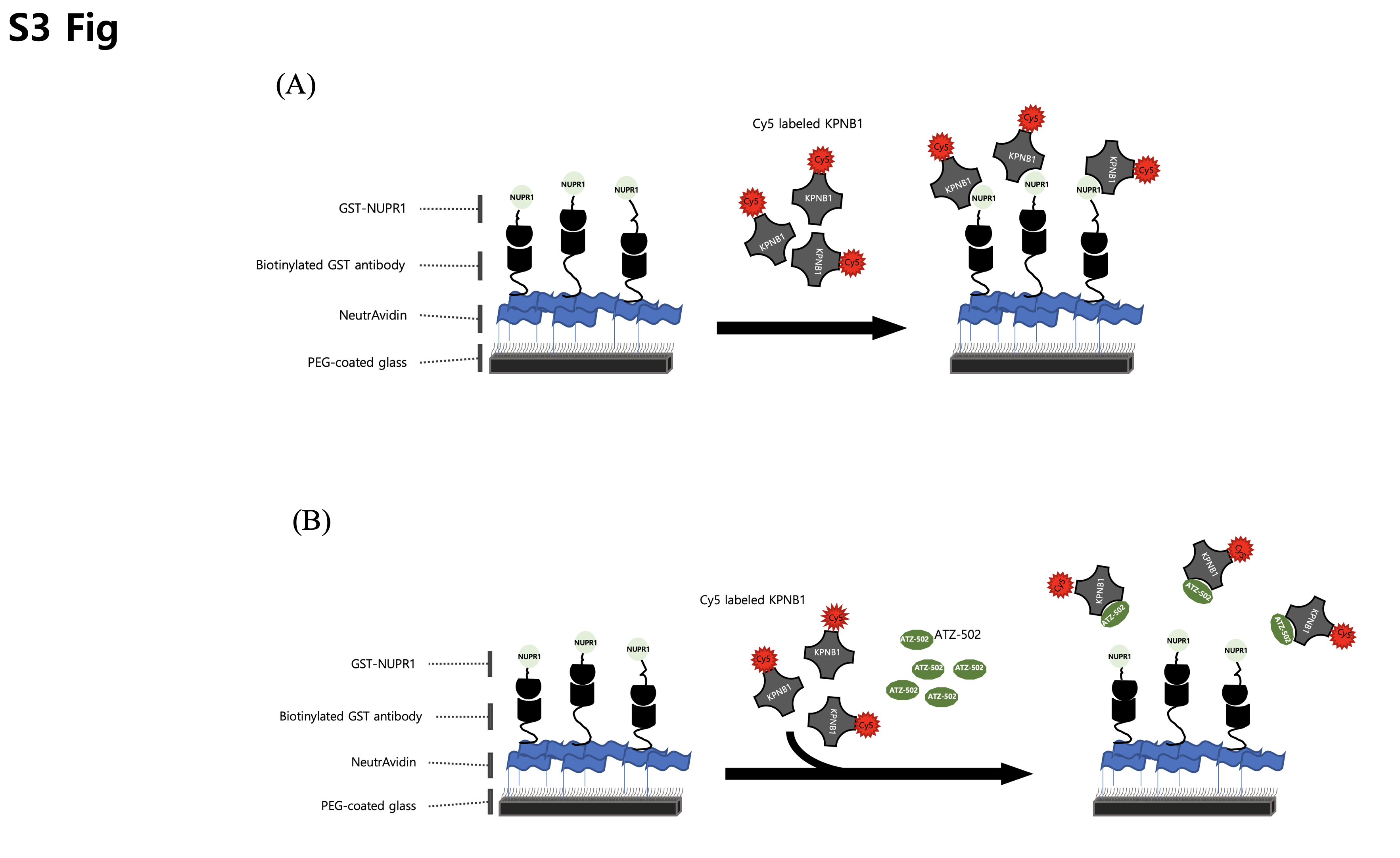


**Supplementary Figure 3**. Illustration of single-molecule binding assay. (A) Schematic illustration of single-molecule binding assay between NUPR1 and KPNB1. (B) Illustration to show the inhibition of the NUPR1 and KPNB1 binding by ATZ-502.


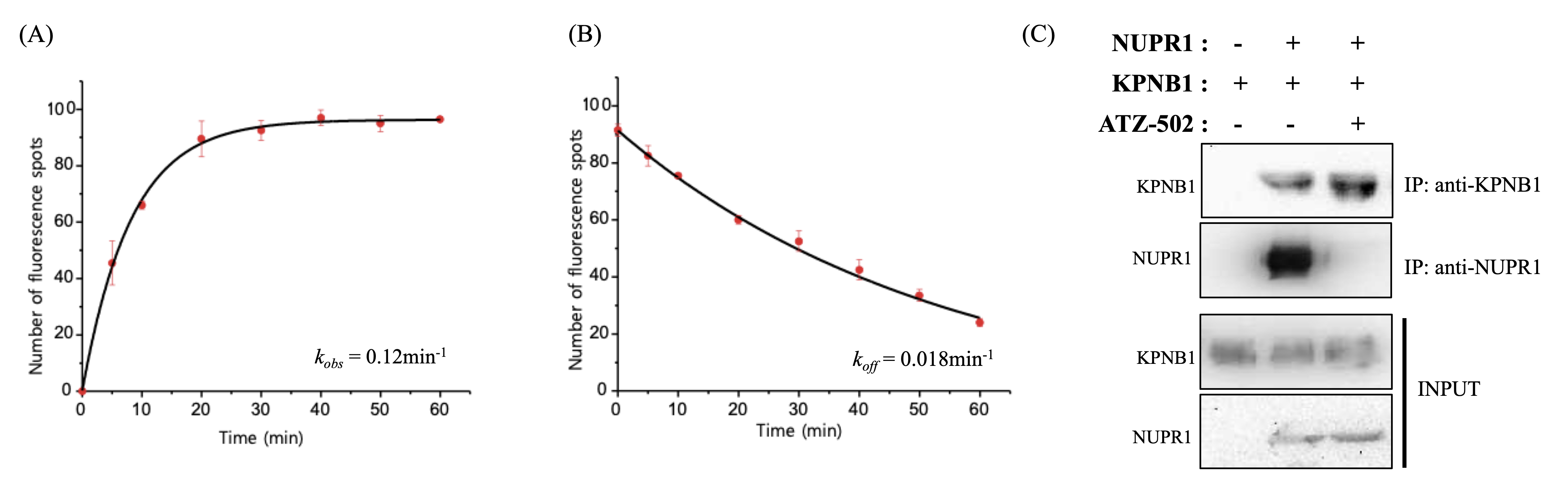


**Supplementary Figure 4.** KPNB1 and NUPR1 binding assay. Using single molecule binding assay (A) The apparent association rate constant, *k_obs_* was obtained by counting the number of NUPR1-bound KPNB1 spots over time upon the addition of 5 nM KPNB1. The association rate constant, *k_on_* was obtained from *k_obs_*= *k_on_* [KPNB1] - *k_off_* which was 0.046 min^-1^ nM^-1^. (B) The dissociation rate constant, *k_off_* was obtained as 0.018 min^-1^ by counting the number of NUPR1-bound KPNB1 spots over time right after removing the freely-diffusing KPNB1 proteins in the solution by substituting the solution with an KPNB1-free imaging buffer. (C) After immunoblotting analysis, NUPR1 (10 nM) protein bind to KPNB1 (10 nM) and these protein binding were inhibited by ATZ-502.

Supplementary Table 1. RNA Quant-seq analysis of doxorubicin-ATZ-502 combination treatment

|  | **Fold change** | | | **Raw data** | | | | **Gene Symbol** | **Description** | **Gene Type** |
| --- | --- | --- | --- | --- | --- | --- | --- | --- | --- | --- |
| **Gene Symbol** | **ATZ-502** | **Doxorubicin** | **Combination** | **Control** | **ATZ-502** | **Doxorubicin** | **Combination** |  |  |  |
| ASF1B | 0.928 | 1.895 | 2.211 | 1200 | 1050 | 2172 | 2828 | ASF1B | ASF1 anti-silencing function 1 homolog B (S. cerevisiae) | protein-coding |
| MYEOV | 0.802 | 0.546 | 0.487 | 4127 | 3124 | 2149 | 2140 | MYEOV | myeloma overexpressed (in a subset of t(11;14) positive multiple myelomas) | protein-coding |
| KTN1 | 1.257 | 1.846 | 2.193 | 1009 | 1197 | 1779 | 2358 | KTN1 | kinectin 1 (kinesin receptor) | protein-coding |
| AKAP2 | 1.152 | 0.519 | 0.494 | 3620 | 3934 | 1792 | 1904 | AKAP2 | A kinase (PRKA) anchor protein 2 | protein-coding |
| SLC20A1 | 0.739 | 0.548 | 0.487 | 2990 | 2084 | 1564 | 1552 | SLC20A1 | solute carrier family 20 (phosphate transporter), member 1 | protein-coding |
| ABT1 | 1.178 | 0.503 | 0.467 | 2121 | 2357 | 1017 | 1055 | ABT1 | activator of basal transcription 1 | protein-coding |
| NDUFB7 | 1.438 | 1.789 | 2.043 | 949 | 1288 | 1622 | 2066 | NDUFB7 | NADH dehydrogenase (ubiquinone) 1 beta subcomplex, 7, 18kDa | protein-coding |
| TOR4A | 0.842 | 0.517 | 0.428 | 1849 | 1469 | 912 | 842 | TOR4A |  |  |
| LMNB1 | 0.847 | 0.567 | 0.495 | 1483 | 1185 | 803 | 782 | LMNB1 | lamin B1 | protein-coding |
| EIF4A2 | 1.316 | 0.643 | 0.498 | 1193 | 1482 | 732 | 633 | EIF4A2 | eukaryotic translation initiation factor 4A2 | protein-coding |
| EMC3-AS1 | 0.925 | 1.864 | 2.546 | 572 | 499 | 1019 | 1553 | EMC3-AS1 |  |  |
| PLK1 | 0.796 | 0.605 | 0.494 | 729 | 547 | 421 | 383 | PLK1 | polo-like kinase 1 | protein-coding |
| RCAN1 | 1.877 | 0.871 | 2.149 | 449 | 796 | 373 | 1029 | RCAN1 | regulator of calcineurin 1 | protein-coding |
| LSG1 | 0.929 | 0.665 | 0.477 | 672 | 589 | 426 | 341 | LSG1 | large subunit GTPase 1 homolog (S. cerevisiae) | protein-coding |
| PDRG1 | 0.888 | 0.506 | 0.496 | 517 | 433 | 249 | 273 | PDRG1 | p53 and DNA-damage regulated 1 | protein-coding |
| PCGF3 | 0.959 | 0.514 | 0.442 | 566 | 512 | 277 | 266 | PCGF3 | polycomb group ring finger 3 | protein-coding |
| BCLAF1 | 1.251 | 1.925 | 2.167 | 443 | 523 | 815 | 1024 | BCLAF1 | BCL2-associated transcription factor 1 | protein-coding |
| C4orf19 | 0.892 | 0.612 | 0.481 | 454 | 382 | 265 | 232 | C4orf19 | chromosome 4 open reading frame 19 | protein-coding |
| WIPF2 | 0.944 | 0.502 | 0.401 | 519 | 462 | 248 | 221 | WIPF2 | WAS/WASL interacting protein family, member 2 | protein-coding |
| FAM20C | 0.580 | 0.565 | 0.489 | 381 | 208 | 205 | 198 | FAM20C |  |  |
| SRSF4 | 0.996 | 0.588 | 0.436 | 414 | 389 | 232 | 192 | SRSF4 | serine/arginine-rich splicing factor 4 | protein-coding |
| JADE2 | 0.544 | 0.563 | 0.430 | 414 | 212 | 222 | 189 | JADE2 |  |  |
| MECP2 | 0.760 | 0.576 | 0.410 | 399 | 286 | 219 | 174 | MECP2 | methyl CpG binding protein 2 (Rett syndrome) | protein-coding |
| SCARNA22 | 1.092 | 1.790 | 2.470 | 429 | 442 | 734 | 1130 | SCARNA22 | small Cajal body-specific RNA 22 | miscRNA |
| PLOD2 | 1.746 | 1.720 | 2.030 | 399 | 658 | 656 | 864 | PLOD2 | procollagen-lysine, 2-oxoglutarate 5-dioxygenase 2 | protein-coding |
| ATRX | 1.341 | 1.675 | 2.281 | 376 | 476 | 602 | 915 | ATRX | alpha thalassemia/mental retardation syndrome X-linked | protein-coding |
| FAM53C | 0.912 | 1.545 | 2.564 | 363 | 312 | 536 | 993 | FAM53C | family with sequence similarity 53, member C | protein-coding |
| ARHGAP11A | 1.241 | 0.509 | 0.394 | 413 | 484 | 200 | 173 | ARHGAP11A | Rho GTPase activating protein 11A | protein-coding |
| TMEM59 | 1.512 | 1.932 | 2.078 | 346 | 494 | 639 | 767 | TMEM59 | transmembrane protein 59 | protein-coding |
| PDCD11 | 0.714 | 0.603 | 0.496 | 315 | 212 | 181 | 166 | PDCD11 | programmed cell death 11 | protein-coding |
| SOX4 | 0.692 | 1.790 | 2.455 | 284 | 185 | 486 | 744 | SOX4 | SRY (sex determining region Y)-box 4 | protein-coding |
| GLS | 1.193 | 1.378 | 2.332 | 262 | 295 | 345 | 652 | GLS | glutaminase | protein-coding |
| SELK | 1.798 | 1.633 | 2.052 | 253 | 430 | 395 | 554 | SELK | selenoprotein K | protein-coding |
| IL6ST | 1.517 | 1.960 | 2.482 | 240 | 344 | 450 | 636 | IL6ST | interleukin 6 signal transducer (gp130, oncostatin M receptor) | protein-coding |
| ATG12 | 1.342 | 1.701 | 2.053 | 236 | 299 | 384 | 517 | ATG12 | ATG12 autophagy related 12 homolog (S. cerevisiae) | protein-coding |
| PPP1R35 | 0.724 | 0.712 | 0.447 | 343 | 234 | 233 | 163 | PPP1R35 |  |  |
| AP1AR | 1.213 | 1.529 | 2.026 | 221 | 253 | 323 | 478 | AP1AR | adaptor-related protein complex 1 associated regulatory protein | protein-coding |
| PPP1R37 | 0.853 | 0.628 | 0.492 | 306 | 246 | 183 | 160 | PPP1R37 |  |  |
| RASSF7 | 0.864 | 0.567 | 0.414 | 350 | 285 | 189 | 154 | RASSF7 | Ras association (RalGDS/AF-6) domain family (N-terminal) member 7 | protein-coding |
| PHIP | 1.431 | 1.224 | 2.019 | 213 | 288 | 249 | 459 | PHIP | pleckstrin homology domain interacting protein | protein-coding |
| NFRKB | 1.222 | 0.549 | 0.456 | 312 | 360 | 163 | 151 | NFRKB | nuclear factor related to kappaB binding protein | protein-coding |
| LINC00472 | 1.134 | 0.537 | 0.429 | 327 | 350 | 167 | 149 | LINC00472 |  |  |
| ARNT | 0.912 | 0.501 | 0.476 | 273 | 235 | 130 | 138 | ARNT | aryl hydrocarbon receptor nuclear translocator | protein-coding |
| BOD1L1 | 1.773 | 1.876 | 2.029 | 210 | 352 | 377 | 455 | BOD1L1 |  |  |
| SNORA12 | 1.023 | 1.984 | 2.860 | 199 | 192 | 378 | 608 | SNORA12 | small nucleolar RNA, H/ACA box 12 | snoRNA |
| NUMBL | 0.855 | 0.558 | 0.476 | 273 | 220 | 145 | 138 | NUMBL | numb homolog (Drosophila)-like | protein-coding |
| SIX1 | 0.938 | 0.516 | 0.365 | 356 | 315 | 175 | 138 | SIX1 | SIX homeobox 1 | protein-coding |
| AMMECR1L | 0.919 | 0.626 | 0.471 | 270 | 234 | 161 | 135 | AMMECR1L | AMME chromosomal region gene 1-like | protein-coding |
| ZGPAT | 1.074 | 0.546 | 0.446 | 283 | 287 | 147 | 134 | ZGPAT | zinc finger, CCCH-type with G patch domain | protein-coding |
| EGR1 | 0.996 | 1.999 | 3.824 | 196 | 184 | 375 | 801 | EGR1 | early growth response 1 | protein-coding |
| PRKAB1 | 0.892 | 0.689 | 0.445 | 277 | 233 | 182 | 131 | PRKAB1 | protein kinase, AMP-activated, beta 1 non-catalytic subunit | protein-coding |
| NCSTN | 1.319 | 1.485 | 2.022 | 195 | 243 | 277 | 421 | NCSTN | nicastrin | protein-coding |
| PIK3C2A | 1.278 | 1.155 | 2.082 | 193 | 233 | 213 | 429 | PIK3C2A | phosphoinositide-3-kinase, class 2, alpha polypeptide | protein-coding |
| ZNF318 | 1.093 | 0.723 | 0.488 | 251 | 259 | 173 | 130 | ZNF318 | zinc finger protein 318 | protein-coding |
| GPSM2 | 1.544 | 0.646 | 0.473 | 257 | 375 | 158 | 129 | GPSM2 | G-protein signaling modulator 2 | protein-coding |
| KLHL12 | 0.675 | 0.552 | 0.433 | 272 | 173 | 143 | 125 | KLHL12 | kelch-like 12 (Drosophila) | protein-coding |
| POMGNT2 | 0.712 | 0.738 | 0.475 | 246 | 165 | 173 | 124 | POMGNT2 |  |  |
| CHST15 | 1.081 | 0.522 | 0.475 | 244 | 249 | 121 | 123 | CHST15 | carbohydrate (N-acetylgalactosamine 4-sulfate 6-O) sulfotransferase 15 | protein-coding |
| GPATCH2L | 1.100 | 1.708 | 2.391 | 186 | 193 | 304 | 475 | GPATCH2L |  |  |
| BROX | 1.101 | 1.705 | 2.019 | 179 | 186 | 292 | 386 | BROX |  |  |
| MAD2L1BP | 1.089 | 0.585 | 0.467 | 248 | 255 | 138 | 123 | MAD2L1BP | MAD2L1 binding protein | protein-coding |
| FAM24B | 1.307 | 1.737 | 2.194 | 175 | 216 | 291 | 410 | FAM24B | family with sequence similarity 24, member B | protein-coding |
| DHX16 | 0.871 | 0.752 | 0.500 | 230 | 189 | 165 | 122 | DHX16 | DEAH (Asp-Glu-Ala-His) box polypeptide 16 | protein-coding |
| BAIAP2 | 0.981 | 1.268 | 2.153 | 160 | 148 | 194 | 368 | BAIAP2 | BAI1-associated protein 2 | protein-coding |
| CAMSAP1 | 0.922 | 0.522 | 0.479 | 238 | 207 | 118 | 121 | CAMSAP1 | calmodulin regulated spectrin-associated protein 1 | protein-coding |
| PDF | 0.976 | 1.001 | 0.498 | 227 | 209 | 217 | 120 | PDF | peptide deformylase (mitochondrial) | protein-coding |
| DNAJB14 | 1.269 | 1.468 | 2.030 | 136 | 163 | 191 | 295 | DNAJB14 | DnaJ (Hsp40) homolog, subfamily B, member 14 | protein-coding |
| COG8 | 0.997 | 0.972 | 0.479 | 236 | 222 | 219 | 120 | COG8 | component of oligomeric golgi complex 8 | protein-coding |
| BAK1 | 0.643 | 0.573 | 0.354 | 317 | 192 | 173 | 119 | BAK1 | BCL2-antagonist/killer 1 | protein-coding |
| IFI27L2 | 1.457 | 1.309 | 2.169 | 135 | 186 | 169 | 313 | IFI27L2 | interferon, alpha-inducible protein 27-like 2 | protein-coding |
| IPMK | 1.487 | 1.737 | 2.316 | 128 | 180 | 213 | 317 | IPMK | inositol polyphosphate multikinase | protein-coding |
| EFNA5 | 1.351 | 0.769 | 0.499 | 217 | 277 | 159 | 115 | EFNA5 | ephrin-A5 | protein-coding |
| RNF41 | 0.852 | 0.732 | 0.494 | 208 | 167 | 145 | 109 | RNF41 | ring finger protein 41 | protein-coding |
| OSBPL8 | 1.830 | 1.898 | 2.370 | 127 | 220 | 231 | 322 | OSBPL8 | oxysterol binding protein-like 8 | protein-coding |
| METTL16 | 1.127 | 0.725 | 0.473 | 217 | 231 | 150 | 109 | METTL16 |  |  |
| RECQL | 1.560 | 1.984 | 2.176 | 122 | 180 | 232 | 284 | RECQL | RecQ protein-like (DNA helicase Q1-like) | protein-coding |
| GPN3 | 1.660 | 1.623 | 2.043 | 119 | 187 | 185 | 260 | GPN3 | GPN-loop GTPase 3 | protein-coding |
| SLC35A3 | 1.636 | 1.470 | 2.069 | 113 | 175 | 159 | 250 | SLC35A3 | solute carrier family 35 (UDP-N-acetylglucosamine (UDP-GlcNAc) transporter), member A3 | protein-coding |
| NCOA6 | 1.155 | 0.525 | 0.319 | 322 | 351 | 161 | 109 | NCOA6 | nuclear receptor coactivator 6 | protein-coding |
| GTF2B | 0.663 | 1.598 | 2.501 | 111 | 69 | 170 | 297 | GTF2B | general transcription factor IIB | protein-coding |
| COG5 | 1.337 | 1.468 | 2.143 | 106 | 134 | 149 | 243 | COG5 | component of oligomeric golgi complex 5 | protein-coding |
| AP5S1 | 0.788 | 0.626 | 0.452 | 225 | 167 | 134 | 108 | AP5S1 |  |  |
| EID2 | 1.054 | 0.725 | 0.487 | 207 | 206 | 143 | 107 | EID2 | EP300 interacting inhibitor of differentiation 2 | protein-coding |
| LRRC14 | 1.044 | 0.556 | 0.485 | 208 | 205 | 110 | 107 | LRRC14 | leucine rich repeat containing 14 | protein-coding |
| MYBBP1A | 0.797 | 0.673 | 0.465 | 217 | 163 | 139 | 107 | MYBBP1A | MYB binding protein (P160) 1a | protein-coding |
| CDCA2 | 0.982 | 0.669 | 0.490 | 204 | 189 | 130 | 106 | CDCA2 | cell division cycle associated 2 | protein-coding |
| PYROXD1 | 1.545 | 1.820 | 2.054 | 106 | 155 | 185 | 233 | PYROXD1 | pyridine nucleotide-disulphide oxidoreductase domain 1 | protein-coding |
| HMGXB4 | 0.991 | 0.539 | 0.406 | 246 | 230 | 126 | 106 | HMGXB4 | HMG box domain containing 4 | protein-coding |
| PIF1 | 1.096 | 0.610 | 0.495 | 200 | 207 | 116 | 105 | PIF1 | PIF1 5'-to-3' DNA helicase homolog (S. cerevisiae) | protein-coding |
| MTPAP | 1.366 | 0.572 | 0.476 | 206 | 266 | 112 | 104 | MTPAP | mitochondrial poly(A) polymerase | protein-coding |
| IP6K1 | 0.869 | 0.566 | 0.478 | 199 | 163 | 107 | 101 | IP6K1 | inositol hexakisphosphate kinase 1 | protein-coding |
| DPF1 | 0.603 | 0.606 | 0.465 | 203 | 115 | 117 | 100 | DPF1 | D4, zinc and double PHD fingers family 1 | protein-coding |
| GIT2 | 0.862 | 0.641 | 0.499 | 187 | 152 | 114 | 99 | GIT2 | G protein-coupled receptor kinase interacting ArfGAP 2 | protein-coding |
| NAB2 | 0.973 | 0.739 | 0.476 | 196 | 180 | 138 | 99 | NAB2 | NGFI-A binding protein 2 (EGR1 binding protein 2) | protein-coding |
| CENPP | 1.054 | 0.658 | 0.447 | 209 | 208 | 131 | 99 | CENPP | centromere protein P | protein-coding |
| F8A1 | 0.730 | 0.574 | 0.389 | 240 | 165 | 131 | 99 | F8A1 | coagulation factor VIII-associated (intronic transcript) 1 | protein-coding |
| LIFR | 1.151 | 1.994 | 2.103 | 104 | 113 | 199 | 234 | LIFR | leukemia inhibitory factor receptor alpha | protein-coding |
| TGFBR1 | 1.721 | 1.659 | 2.177 | 100 | 163 | 159 | 233 | TGFBR1 | transforming growth factor, beta receptor 1 | protein-coding |
| CDKN1B | 1.119 | 0.639 | 0.476 | 194 | 205 | 118 | 98 | CDKN1B | cyclin-dependent kinase inhibitor 1B (p27, Kip1) | protein-coding |
| RNF168 | 0.756 | 0.616 | 0.479 | 191 | 136 | 112 | 97 | RNF168 | ring finger protein 168 | protein-coding |
| ZNF721 | 1.221 | 1.386 | 2.145 | 98 | 113 | 130 | 225 | ZNF721 | zinc finger protein 721 | protein-coding |
| IPPK | 1.019 | 0.656 | 0.440 | 208 | 200 | 130 | 97 | IPPK | inositol 1,3,4,5,6-pentakisphosphate 2-kinase | protein-coding |
| ANKRD12 | 1.763 | 1.796 | 2.972 | 97 | 162 | 167 | 309 | ANKRD12 | ankyrin repeat domain 12 | protein-coding |
| KDM2B | 0.894 | 0.556 | 0.410 | 223 | 188 | 118 | 97 | KDM2B | lysine (K)-specific demethylase 2B | protein-coding |
| TRIP11 | 1.805 | 1.197 | 2.349 | 97 | 166 | 111 | 244 | TRIP11 | thyroid hormone receptor interactor 11 | protein-coding |
| TCHP | 0.910 | 0.609 | 0.401 | 226 | 194 | 131 | 96 | TCHP | trichoplein, keratin filament binding | protein-coding |
| KCNN4 | 0.769 | 0.704 | 0.484 | 185 | 134 | 124 | 95 | KCNN4 | potassium intermediate/small conductance calcium-activated channel, subfamily N, member 4 | protein-coding |
| UBE2D1 | 1.005 | 1.614 | 2.183 | 95 | 90 | 147 | 222 | UBE2D1 | ubiquitin-conjugating enzyme E2D 1 (UBC4/5 homolog, yeast) | protein-coding |
| YPEL5 | 1.016 | 1.712 | 2.330 | 95 | 91 | 156 | 237 | YPEL5 | yippee-like 5 (Drosophila) | protein-coding |
| SSX2IP | 1.254 | 1.250 | 2.355 | 92 | 109 | 110 | 232 | SSX2IP | synovial sarcoma, X breakpoint 2 interacting protein | protein-coding |
| MAML1 | 0.890 | 0.516 | 0.435 | 206 | 173 | 101 | 95 | MAML1 | mastermind-like 1 (Drosophila) | protein-coding |
| PAG1 | 0.790 | 0.561 | 0.482 | 180 | 134 | 96 | 92 | PAG1 | phosphoprotein associated with glycosphingolipid microdomains 1 | protein-coding |
| STK10 | 0.721 | 0.511 | 0.357 | 243 | 165 | 118 | 92 | STK10 | serine/threonine kinase 10 | protein-coding |
| FOXJ3 | 0.849 | 0.625 | 0.485 | 175 | 140 | 104 | 90 | FOXJ3 | forkhead box J3 | protein-coding |
| KMT2E | 1.075 | 0.674 | 0.429 | 198 | 201 | 127 | 90 | KMT2E |  |  |
| SNRNP35 | 1.055 | 0.741 | 0.338 | 249 | 248 | 176 | 89 | SNRNP35 | small nuclear ribonucleoprotein 35kDa (U11/U12) | protein-coding |
| CD24 | 1.339 | 1.795 | 2.086 | 90 | 114 | 155 | 201 | CD24 | CD24 molecule | protein-coding |
| CENPK | 1.334 | 1.530 | 2.059 | 88 | 111 | 129 | 194 | CENPK | centromere protein K | protein-coding |
| MAPK9 | 1.714 | 1.647 | 2.006 | 88 | 143 | 139 | 189 | MAPK9 | mitogen-activated protein kinase 9 | protein-coding |
| ODF2L | 1.417 | 1.571 | 2.076 | 85 | 114 | 128 | 189 | ODF2L | outer dense fiber of sperm tails 2-like | protein-coding |
| HBP1 | 1.097 | 1.232 | 2.024 | 84 | 87 | 99 | 182 | HBP1 | HMG-box transcription factor 1 | protein-coding |
| FAM160A2 | 1.084 | 0.579 | 0.485 | 171 | 175 | 94 | 88 | FAM160A2 | family with sequence similarity 160, member A2 | protein-coding |
| CCDC84 | 0.868 | 0.585 | 0.439 | 187 | 153 | 104 | 87 | CCDC84 | coiled-coil domain containing 84 | protein-coding |
| POMGNT1 | 1.259 | 1.725 | 2.367 | 84 | 100 | 139 | 213 | POMGNT1 | protein O-linked mannose beta1,2-N-acetylglucosaminyltransferase | protein-coding |
| ZFYVE27 | 0.859 | 0.792 | 0.498 | 163 | 132 | 123 | 86 | ZFYVE27 | zinc finger, FYVE domain containing 27 | protein-coding |
| NEURL4 | 1.064 | 0.502 | 0.346 | 235 | 236 | 112 | 86 | NEURL4 | neuralized homolog 4 (Drosophila) | protein-coding |
| CYB561D1 | 0.958 | 0.586 | 0.381 | 208 | 188 | 116 | 84 | CYB561D1 | cytochrome b-561 domain containing 1 | protein-coding |
| RASA3 | 0.937 | 0.571 | 0.290 | 267 | 236 | 145 | 82 | RASA3 | RAS p21 protein activator 3 | protein-coding |
| TACC2 | 1.018 | 0.531 | 0.499 | 153 | 147 | 77 | 81 | TACC2 | transforming, acidic coiled-coil containing protein 2 | protein-coding |
| SNAPC4 | 0.685 | 0.506 | 0.432 | 177 | 114 | 85 | 81 | SNAPC4 | small nuclear RNA activating complex, polypeptide 4, 190kDa | protein-coding |
| PDCD2L | 0.854 | 0.723 | 0.490 | 154 | 124 | 106 | 80 | PDCD2L | programmed cell death 2-like | protein-coding |
| PFAS | 0.670 | 0.777 | 0.490 | 154 | 97 | 114 | 80 | PFAS | phosphoribosylformylglycinamidine synthase | protein-coding |
| GSTCD | 0.983 | 1.481 | 2.098 | 81 | 75 | 115 | 182 | GSTCD | glutathione S-transferase, C-terminal domain containing | protein-coding |
| BMPR2 | 1.060 | 1.884 | 2.080 | 79 | 79 | 143 | 176 | BMPR2 | bone morphogenetic protein receptor, type II (serine/threonine kinase) | protein-coding |
| C21orf91 | 1.246 | 1.898 | 2.808 | 79 | 93 | 144 | 238 | C21orf91 | chromosome 21 open reading frame 91 | protein-coding |
| RP9 | 1.265 | 0.635 | 0.460 | 164 | 196 | 99 | 80 | RP9 | retinitis pigmentosa 9 (autosomal dominant) | protein-coding |
| RGAG4 | 0.591 | 1.603 | 2.047 | 78 | 43 | 120 | 171 | RGAG4 | retrotransposon gag domain containing 4 | protein-coding |
| LRP5 | 0.502 | 0.691 | 0.494 | 149 | 70 | 98 | 78 | LRP5 | low density lipoprotein receptor-related protein 5 | protein-coding |
| PLEKHA1 | 1.211 | 1.075 | 2.259 | 76 | 87 | 78 | 184 | PLEKHA1 | pleckstrin homology domain containing, family A (phosphoinositide binding specific) member 1 | protein-coding |
| AGAP1 | 0.883 | 0.575 | 0.440 | 161 | 134 | 88 | 75 | AGAP1 | ArfGAP with GTPase domain, ankyrin repeat and PH domain 1 | protein-coding |
| ITGAV | 1.367 | 1.832 | 2.597 | 75 | 97 | 132 | 209 | ITGAV | integrin, alpha V (vitronectin receptor, alpha polypeptide, antigen CD51) | protein-coding |
| RPS6KA5 | 1.498 | 1.564 | 2.369 | 74 | 105 | 111 | 188 | RPS6KA5 | ribosomal protein S6 kinase, 90kDa, polypeptide 5 | protein-coding |
| PID1 | 0.818 | 0.632 | 0.382 | 183 | 141 | 110 | 74 | PID1 | phosphotyrosine interaction domain containing 1 | protein-coding |
| RNMTL1 | 0.987 | 0.570 | 0.472 | 146 | 136 | 79 | 73 | RNMTL1 | RNA methyltransferase like 1 | protein-coding |
| APBB2 | 1.197 | 0.816 | 0.451 | 153 | 173 | 119 | 73 | APBB2 | amyloid beta (A4) precursor protein-binding, family B, member 2 | protein-coding |
| REV1 | 1.303 | 1.415 | 2.058 | 73 | 90 | 99 | 161 | REV1 | REV1 homolog (S. cerevisiae) | protein-coding |
| TMTC3 | 1.647 | 1.811 | 2.350 | 73 | 114 | 127 | 184 | TMTC3 | transmembrane and tetratricopeptide repeat containing 3 | protein-coding |
| C9orf142 | 1.640 | 1.205 | 2.060 | 72 | 112 | 83 | 159 | C9orf142 | chromosome 9 open reading frame 142 | protein-coding |
| IDI1 | 1.641 | 1.710 | 2.118 | 70 | 109 | 115 | 159 | IDI1 | isopentenyl-diphosphate delta isomerase 1 | protein-coding |
| E2F4 | 1.128 | 0.544 | 0.370 | 184 | 196 | 95 | 72 | E2F4 | E2F transcription factor 4, p107/p130-binding | protein-coding |
| PNPLA8 | 1.328 | 1.401 | 2.159 | 70 | 88 | 94 | 162 | PNPLA8 | patatin-like phospholipase domain containing 8 | protein-coding |
| MB21D2 | 0.662 | 0.816 | 0.497 | 135 | 84 | 105 | 71 | MB21D2 |  |  |
| PGM2L1 | 1.695 | 1.526 | 2.000 | 69 | 111 | 101 | 148 | PGM2L1 | phosphoglucomutase 2-like 1 | protein-coding |
| SHQ1 | 0.550 | 0.549 | 0.365 | 184 | 95 | 96 | 71 | SHQ1 | SHQ1 homolog (S. cerevisiae) | protein-coding |
| FAM213B | 1.613 | 1.806 | 2.056 | 68 | 104 | 118 | 150 | FAM213B |  |  |
| FAM76B | 1.316 | 0.794 | 2.152 | 65 | 81 | 49 | 150 | FAM76B | family with sequence similarity 76, member B | protein-coding |
| ACADSB | 1.728 | 1.611 | 2.010 | 64 | 105 | 99 | 138 | ACADSB | acyl-CoA dehydrogenase, short/branched chain | protein-coding |
| TBC1D9 | 1.622 | 1.734 | 2.321 | 63 | 97 | 105 | 157 | TBC1D9 | TBC1 domain family, member 9 (with GRAM domain) | protein-coding |
| BTRC | 0.787 | 0.538 | 0.437 | 147 | 109 | 75 | 68 | BTRC | beta-transducin repeat containing | protein-coding |
| FMNL3 | 0.617 | 0.542 | 0.459 | 138 | 80 | 71 | 67 | FMNL3 | formin-like 3 | protein-coding |
| XYLT2 | 1.086 | 0.694 | 0.406 | 156 | 160 | 103 | 67 | XYLT2 | xylosyltransferase II | protein-coding |
| EFCAB11 | 1.197 | 1.705 | 2.229 | 61 | 69 | 100 | 146 | EFCAB11 |  |  |
| TRIT1 | 1.092 | 0.740 | 0.499 | 125 | 129 | 88 | 66 | TRIT1 | tRNA isopentenyltransferase 1 | protein-coding |
| ESPL1 | 0.706 | 0.598 | 0.491 | 125 | 83 | 71 | 65 | ESPL1 | extra spindle pole bodies homolog 1 (S. cerevisiae) | protein-coding |
| STS | 1.529 | 1.785 | 2.019 | 60 | 87 | 103 | 130 | STS | steroid sulfatase (microsomal), isozyme S | protein-coding |
| TM2D3 | 1.003 | 0.823 | 0.465 | 130 | 123 | 102 | 64 | TM2D3 | TM2 domain containing 3 | protein-coding |
| ING5 | 1.169 | 0.626 | 0.423 | 143 | 158 | 85 | 64 | ING5 | inhibitor of growth family, member 5 | protein-coding |
| RFWD2 | 0.867 | 0.709 | 0.473 | 126 | 103 | 85 | 63 | RFWD2 | ring finger and WD repeat domain 2 | protein-coding |
| TMEM138 | 1.059 | 0.513 | 0.426 | 140 | 140 | 68 | 63 | TMEM138 | transmembrane protein 138 | protein-coding |
| ARHGEF2 | 1.575 | 0.511 | 0.488 | 120 | 179 | 58 | 62 | ARHGEF2 | Rho/Rac guanine nucleotide exchange factor (GEF) 2 | protein-coding |
| DDIAS | 1.113 | 1.100 | 2.057 | 58 | 61 | 61 | 128 | DDIAS |  |  |
| CAPRIN2 | 1.067 | 0.798 | 0.469 | 125 | 126 | 95 | 62 | CAPRIN2 | caprin family member 2 | protein-coding |
| CHMP4C | 1.735 | 0.921 | 2.156 | 57 | 94 | 50 | 132 | CHMP4C | chromatin modifying protein 4C | protein-coding |
| RP2 | 1.480 | 1.841 | 2.529 | 57 | 80 | 101 | 155 | RP2 | retinitis pigmentosa 2 (X-linked recessive) | protein-coding |
| EPHA4 | 1.060 | 1.541 | 2.035 | 54 | 54 | 80 | 118 | EPHA4 | EPH receptor A4 | protein-coding |
| GCLC | 1.491 | 1.086 | 2.195 | 53 | 75 | 55 | 125 | GCLC | glutamate-cysteine ligase, catalytic subunit | protein-coding |
| MYLIP | 1.080 | 1.570 | 2.527 | 53 | 54 | 80 | 144 | MYLIP | myosin regulatory light chain interacting protein | protein-coding |
| DCLRE1B | 0.894 | 0.602 | 0.412 | 140 | 118 | 80 | 61 | DCLRE1B | DNA cross-link repair 1B | protein-coding |
| PXN-AS1 | 1.079 | 1.686 | 2.021 | 53 | 54 | 86 | 115 | PXN-AS1 |  |  |
| PPCDC | 0.620 | 0.689 | 0.465 | 122 | 71 | 80 | 60 | PPCDC | phosphopantothenoylcysteine decarboxylase | protein-coding |
| ZNRF3 | 1.408 | 0.619 | 0.489 | 114 | 152 | 67 | 59 | ZNRF3 | zinc and ring finger 3 | protein-coding |
| CLU | 0.755 | 1.730 | 2.063 | 51 | 36 | 85 | 113 | CLU | clusterin | protein-coding |
| ADAT2 | 1.187 | 0.668 | 0.477 | 115 | 129 | 73 | 58 | ADAT2 | adenosine deaminase, tRNA-specific 2, TAD2 homolog (S. cerevisiae) | protein-coding |
| HES1 | 0.831 | 0.883 | 2.123 | 50 | 39 | 42 | 114 | HES1 | hairy and enhancer of split 1, (Drosophila) | protein-coding |
| NUDT19 | 0.947 | 0.681 | 0.450 | 122 | 109 | 79 | 58 | NUDT19 | nudix (nucleoside diphosphate linked moiety X)-type motif 19 | protein-coding |
| APCDD1L | 0.601 | 0.820 | 0.490 | 110 | 62 | 86 | 57 | APCDD1L | adenomatosis polyposis coli down-regulated 1-like | protein-coding |
| MIR6805 | 1.371 | 1.232 | 2.214 | 50 | 65 | 59 | 119 | MIR6805 |  |  |
| BRF1 | 0.979 | 0.572 | 0.457 | 118 | 109 | 64 | 57 | BRF1 | BRF1 homolog, subunit of RNA polymerase III transcription initiation factor IIIB (S. cerevisiae) | protein-coding |
| INHBB | 0.672 | 0.751 | 0.375 | 144 | 91 | 103 | 57 | INHBB | inhibin, beta B | protein-coding |
| CENPQ | 1.991 | 1.780 | 2.162 | 49 | 93 | 84 | 114 | CENPQ | centromere protein Q | protein-coding |
| CDCA7 | 0.911 | 0.561 | 0.245 | 221 | 190 | 118 | 57 | CDCA7 | cell division cycle associated 7 | protein-coding |
| LINC00342 | 0.848 | 0.629 | 2.184 | 49 | 39 | 29 | 115 | LINC00342 |  |  |
| C3orf38 | 0.920 | 0.634 | 0.469 | 113 | 98 | 68 | 56 | C3orf38 | chromosome 3 open reading frame 38 | protein-coding |
| CDC42EP4 | 0.901 | 0.646 | 0.491 | 106 | 90 | 65 | 55 | CDC42EP4 | CDC42 effector protein (Rho GTPase binding) 4 | protein-coding |
| RICTOR | 1.315 | 1.989 | 2.973 | 49 | 61 | 94 | 157 | RICTOR | RPTOR independent companion of MTOR, complex 2 | protein-coding |
| HEATR6 | 1.100 | 0.846 | 0.404 | 129 | 134 | 104 | 55 | HEATR6 | HEAT repeat containing 6 | protein-coding |
| UCN | 1.187 | 1.403 | 2.164 | 49 | 55 | 66 | 114 | UCN | urocortin | protein-coding |
| AHI1 | 1.579 | 1.539 | 2.610 | 48 | 72 | 71 | 135 | AHI1 | Abelson helper integration site 1 | protein-coding |
| GPN2 | 0.547 | 0.957 | 0.403 | 127 | 65 | 116 | 54 | GPN2 | GPN-loop GTPase 2 | protein-coding |
| LRRC8D | 1.069 | 0.925 | 0.492 | 102 | 103 | 90 | 53 | LRRC8D | leucine rich repeat containing 8 family, member D | protein-coding |
| DIP2A | 1.078 | 0.821 | 0.456 | 110 | 112 | 86 | 53 | DIP2A | DIP2 disco-interacting protein 2 homolog A (Drosophila) | protein-coding |
| DCAF4 | 1.076 | 0.627 | 0.415 | 121 | 123 | 72 | 53 | DCAF4 | DDB1 and CUL4 associated factor 4 | protein-coding |
| MTSS1L | 0.787 | 0.608 | 0.473 | 104 | 77 | 60 | 52 | MTSS1L | metastasis suppressor 1-like | protein-coding |
| GBP2 | 1.859 | 1.539 | 2.379 | 48 | 85 | 71 | 123 | GBP2 | guanylate binding protein 2, interferon-inducible | protein-coding |
| HSCB | 1.363 | 1.837 | 2.226 | 48 | 62 | 85 | 115 | HSCB | HscB iron-sulfur cluster co-chaperone homolog (E. coli) | protein-coding |
| GPR176 | 1.069 | 0.593 | 0.492 | 98 | 99 | 55 | 51 | GPR176 | G protein-coupled receptor 176 | protein-coding |
| CNOT10 | 1.207 | 0.649 | 0.488 | 99 | 113 | 61 | 51 | CNOT10 | CCR4-NOT transcription complex, subunit 10 | protein-coding |
| IFI6 | 0.585 | 1.815 | 2.189 | 48 | 26 | 84 | 113 | IFI6 | interferon, alpha-inducible protein 6 | protein-coding |
| MLH3 | 0.884 | 0.569 | 0.474 | 102 | 85 | 55 | 51 | MLH3 | mutL homolog 3 (E. coli) | protein-coding |
| ANKRD10 | 1.051 | 0.863 | 0.390 | 124 | 123 | 102 | 51 | ANKRD10 | ankyrin repeat domain 10 | protein-coding |
| CHAF1B | 0.551 | 0.620 | 0.321 | 151 | 78 | 89 | 51 | CHAF1B | chromatin assembly factor 1, subunit B (p60) | protein-coding |
| JRKL | 1.170 | 0.778 | 0.455 | 104 | 115 | 77 | 50 | JRKL | jerky homolog-like (mouse) | protein-coding |
| LDOC1 | 0.876 | 1.615 | 2.066 | 45 | 37 | 70 | 100 | LDOC1 | leucine zipper, down-regulated in cancer 1 | protein-coding |
| LIMK1 | 0.709 | 0.653 | 0.439 | 108 | 72 | 67 | 50 | LIMK1 | LIM domain kinase 1 | protein-coding |
| NIT1 | 0.508 | 0.842 | 0.389 | 122 | 58 | 98 | 50 | NIT1 | nitrilase 1 | protein-coding |
| MBNL2 | 1.037 | 1.070 | 2.743 | 45 | 44 | 46 | 133 | MBNL2 | muscleblind-like 2 (Drosophila) | protein-coding |
| ANKS3 | 1.154 | 0.571 | 0.329 | 144 | 157 | 78 | 50 | ANKS3 | ankyrin repeat and sterile alpha motif domain containing 3 | protein-coding |
| VPS50 | 1.796 | 1.548 | 2.003 | 45 | 77 | 67 | 97 | VPS50 |  |  |
| TPST2 | 0.981 | 1.179 | 0.493 | 94 | 87 | 106 | 49 | TPST2 | tyrosylprotein sulfotransferase 2 | protein-coding |
| CMTM3 | 1.026 | 0.654 | 0.488 | 95 | 92 | 59 | 49 | CMTM3 | CKLF-like MARVEL transmembrane domain containing 3 | protein-coding |
| POLR3E | 1.134 | 0.534 | 0.478 | 97 | 104 | 49 | 49 | POLR3E | polymerase (RNA) III (DNA directed) polypeptide E (80kD) | protein-coding |
| CDC42EP1 | 0.920 | 0.519 | 0.474 | 98 | 85 | 48 | 49 | CDC42EP1 | CDC42 effector protein (Rho GTPase binding) 1 | protein-coding |
| GNB1L | 0.712 | 0.902 | 0.404 | 115 | 77 | 99 | 49 | GNB1L | guanine nucleotide binding protein (G protein), beta polypeptide 1-like | protein-coding |
| MED17 | 1.682 | 0.739 | 0.394 | 118 | 188 | 83 | 49 | MED17 | mediator complex subunit 17 | protein-coding |
| EN2 | 1.060 | 1.906 | 2.343 | 44 | 44 | 81 | 111 | EN2 | engrailed homeobox 2 | protein-coding |
| AFAP1L2 | 0.936 | 0.722 | 0.363 | 128 | 113 | 88 | 49 | AFAP1L2 | actin filament associated protein 1-like 2 | protein-coding |
| SPSB2 | 1.234 | 0.614 | 0.352 | 132 | 154 | 77 | 49 | SPSB2 | splA/ryanodine receptor domain and SOCS box containing 2 | protein-coding |
| LMBRD2 | 0.916 | 1.808 | 2.460 | 43 | 37 | 75 | 114 | LMBRD2 | LMBR1 domain containing 2 | protein-coding |
| C4orf32 | 1.798 | 1.510 | 2.340 | 42 | 72 | 61 | 106 | C4orf32 | chromosome 4 open reading frame 32 | protein-coding |
| C17orf58 | 1.093 | 0.915 | 0.483 | 94 | 97 | 82 | 48 | C17orf58 | chromosome 17 open reading frame 58 | protein-coding |
| DOLK | 0.902 | 0.664 | 0.455 | 100 | 85 | 63 | 48 | DOLK | dolichol kinase | protein-coding |
| RAB30 | 1.488 | 0.742 | 0.469 | 95 | 134 | 67 | 47 | RAB30 | RAB30, member RAS oncogene family | protein-coding |
| SGMS1 | 1.058 | 0.567 | 0.469 | 95 | 95 | 51 | 47 | SGMS1 | sphingomyelin synthase 1 | protein-coding |
| RSBN1L | 1.306 | 0.869 | 0.459 | 93 | 115 | 77 | 45 | RSBN1L | round spermatid basic protein 1-like | protein-coding |
| GPRASP2 | 0.760 | 0.751 | 0.496 | 84 | 60 | 60 | 44 | GPRASP2 | G protein-coupled receptor associated sorting protein 2 | protein-coding |
| DSEL | 0.854 | 1.226 | 2.043 | 40 | 32 | 47 | 88 | DSEL | dermatan sulfate epimerase-like | protein-coding |
| SAMD10 | 0.536 | 0.741 | 0.474 | 88 | 44 | 62 | 44 | SAMD10 | sterile alpha motif domain containing 10 | protein-coding |
| TMCO3 | 1.214 | 0.920 | 2.114 | 40 | 46 | 35 | 91 | TMCO3 | transmembrane and coiled-coil domains 3 | protein-coding |
| ABHD14A | 0.743 | 1.126 | 2.000 | 39 | 27 | 42 | 84 | ABHD14A | abhydrolase domain containing 14A | protein-coding |
| PROSER1 | 1.108 | 0.783 | 0.394 | 106 | 111 | 79 | 44 | PROSER1 |  |  |
| ZBED4 | 1.106 | 0.524 | 0.376 | 111 | 116 | 55 | 44 | ZBED4 | zinc finger, BED-type containing 4 | protein-coding |
| BET1 | 1.928 | 1.316 | 2.316 | 38 | 70 | 48 | 95 | BET1 | blocked early in transport 1 homolog (S. cerevisiae) | protein-coding |
| RGS2 | 0.924 | 1.476 | 2.052 | 38 | 33 | 54 | 84 | RGS2 | regulator of G-protein signaling 2, 24kDa | protein-coding |
| CCDC82 | 1.701 | 1.158 | 2.749 | 37 | 60 | 41 | 110 | CCDC82 | coiled-coil domain containing 82 | protein-coding |
| CMBL | 0.946 | 1.330 | 2.086 | 36 | 32 | 46 | 81 | CMBL | carboxymethylenebutenolidase homolog (Pseudomonas) | protein-coding |
| CSNK2A3 | 0.917 | 0.868 | 0.492 | 81 | 70 | 67 | 42 | CSNK2A3 |  |  |
| LMNTD2 | 0.943 | 1.338 | 2.145 | 35 | 31 | 45 | 81 | LMNTD2 |  |  |
| CD27-AS1 | 0.788 | 1.345 | 2.181 | 34 | 25 | 44 | 80 | CD27-AS1 |  |  |
| FXN | 0.901 | 1.058 | 0.429 | 93 | 79 | 94 | 42 | FXN | frataxin | protein-coding |
| KLHL28 | 1.997 | 1.407 | 2.662 | 34 | 65 | 46 | 98 | KLHL28 | kelch-like 28 (Drosophila) | protein-coding |
| TMEM254 | 0.557 | 0.906 | 0.415 | 96 | 50 | 83 | 42 | TMEM254 |  |  |
| FANCC | 0.929 | 0.694 | 0.411 | 97 | 85 | 64 | 42 | FANCC | Fanconi anemia, complementation group C | protein-coding |
| C22orf15 | 0.520 | 0.839 | 0.380 | 105 | 51 | 84 | 42 | C22orf15 | chromosome 22 open reading frame 15 | protein-coding |
| FKBPL | 1.411 | 0.556 | 0.486 | 80 | 107 | 42 | 41 | FKBPL | FK506 binding protein like | protein-coding |
| MOCOS | 1.181 | 0.803 | 0.458 | 85 | 95 | 65 | 41 | MOCOS | molybdenum cofactor sulfurase | protein-coding |
| CABLES2 | 0.832 | 0.812 | 0.402 | 97 | 76 | 75 | 41 | CABLES2 | Cdk5 and Abl enzyme substrate 2 | protein-coding |
| LMBR1L | 0.667 | 0.737 | 0.475 | 80 | 50 | 56 | 40 | LMBR1L | limb region 1 homolog (mouse)-like | protein-coding |
| CHAMP1 | 1.564 | 0.933 | 0.417 | 91 | 135 | 81 | 40 | CHAMP1 |  |  |
| ZCCHC11 | 0.919 | 0.716 | 0.494 | 75 | 65 | 51 | 39 | ZCCHC11 | zinc finger, CCHC domain containing 11 | protein-coding |
| ROBO4 | 0.739 | 0.560 | 0.436 | 85 | 59 | 45 | 39 | ROBO4 | roundabout homolog 4, magic roundabout (Drosophila) | protein-coding |
| LRRC56 | 1.000 | 1.376 | 2.179 | 34 | 32 | 45 | 80 | LRRC56 | leucine rich repeat containing 56 | protein-coding |
| CTSS | 1.217 | 1.418 | 2.434 | 33 | 38 | 45 | 87 | CTSS | cathepsin S | protein-coding |
| UBQLN2 | 0.828 | 1.455 | 0.431 | 86 | 67 | 120 | 39 | UBQLN2 | ubiquilin 2 | protein-coding |
| PRDM15 | 0.749 | 0.529 | 0.421 | 88 | 62 | 44 | 39 | PRDM15 | PR domain containing 15 | protein-coding |
| MAGEF1 | 1.402 | 0.569 | 0.248 | 150 | 199 | 81 | 39 | MAGEF1 | melanoma antigen family F, 1 | protein-coding |
| PTCH2 | 0.687 | 0.608 | 0.494 | 73 | 47 | 42 | 38 | PTCH2 | patched 2 | protein-coding |
| REV3L | 1.651 | 0.987 | 2.853 | 33 | 52 | 31 | 102 | REV3L | REV3-like, catalytic subunit of DNA polymerase zeta (yeast) | protein-coding |
| TMSB15B | 1.124 | 1.839 | 2.197 | 32 | 34 | 57 | 76 | TMSB15B | thymosin beta 15B | protein-coding |
| BMS1P20 | 1.028 | 1.962 | 2.264 | 31 | 30 | 59 | 76 | BMS1P20 |  |  |
| CEP19 | 1.390 | 1.080 | 2.090 | 31 | 41 | 32 | 70 | CEP19 |  |  |
| SNX18 | 0.701 | 0.719 | 0.457 | 79 | 52 | 54 | 38 | SNX18 | sorting nexin 18 | protein-coding |
| FCGRT | 1.127 | 1.766 | 2.707 | 31 | 33 | 53 | 91 | FCGRT | Fc fragment of IgG, receptor, transporter, alpha | protein-coding |
| CRAT | 1.742 | 1.385 | 2.338 | 30 | 50 | 40 | 76 | CRAT | carnitine O-acetyltransferase | protein-coding |
| SERINC5 | 0.923 | 1.586 | 2.097 | 30 | 26 | 46 | 68 | SERINC5 | serine incorporator 5 | protein-coding |
| CRISPLD2 | 1.801 | 1.919 | 3.328 | 29 | 50 | 54 | 105 | CRISPLD2 | cysteine-rich secretory protein LCCL domain containing 2 | protein-coding |
| CENPT | 0.613 | 0.643 | 0.441 | 82 | 47 | 50 | 38 | CENPT | centromere protein T | protein-coding |
| EVC | 0.702 | 0.645 | 0.425 | 85 | 56 | 52 | 38 | EVC | Ellis van Creveld syndrome | protein-coding |
| FYCO1 | 0.847 | 0.642 | 0.475 | 74 | 59 | 45 | 37 | FYCO1 | FYVE and coiled-coil domain containing 1 | protein-coding |
| CHST2 | 0.911 | 0.953 | 0.451 | 78 | 67 | 71 | 37 | CHST2 | carbohydrate (N-acetylglucosamine-6-O) sulfotransferase 2 | protein-coding |
| CTSH | 1.166 | 1.918 | 2.165 | 29 | 32 | 54 | 68 | CTSH | cathepsin H | protein-coding |
| ESRP2 | 1.200 | 0.651 | 0.434 | 81 | 92 | 50 | 37 | ESRP2 | epithelial splicing regulatory protein 2 | protein-coding |
| KRCC1 | 1.248 | 1.718 | 2.356 | 27 | 32 | 45 | 69 | KRCC1 | lysine-rich coiled-coil 1 | protein-coding |
| ZBTB24 | 1.174 | 0.656 | 0.429 | 82 | 91 | 51 | 37 | ZBTB24 | zinc finger and BTB domain containing 24 | protein-coding |
| PP7080 | 1.013 | 0.642 | 0.383 | 92 | 88 | 56 | 37 | PP7080 |  |  |
| LAMTOR3 | 1.363 | 1.795 | 3.163 | 27 | 35 | 47 | 93 | LAMTOR3 |  |  |
| ZNF252P | 0.706 | 0.833 | 0.383 | 92 | 61 | 73 | 37 | ZNF252P |  |  |
| PABPC1L | 1.438 | 1.682 | 2.388 | 27 | 37 | 44 | 70 | PABPC1L | poly(A) binding protein, cytoplasmic 1-like | protein-coding |
| SPON2 | 0.457 | 1.981 | 2.657 | 27 | 11 | 52 | 78 | SPON2 |  |  |
| DNM1 | 1.374 | 2.054 | 2.617 | 26 | 34 | 52 | 74 | DNM1 | dynamin 1 | protein-coding |
| MBNL3 | 1.061 | 1.860 | 2.722 | 26 | 26 | 47 | 77 | MBNL3 | muscleblind-like 3 (Drosophila) | protein-coding |
| BAG4 | 1.614 | 0.825 | 0.433 | 79 | 121 | 62 | 36 | BAG4 | BCL2-associated athanogene 4 | protein-coding |
| NDST2 | 0.936 | 0.791 | 0.403 | 85 | 75 | 64 | 36 | NDST2 | N-deacetylase/N-sulfotransferase (heparan glucosaminyl) 2 | protein-coding |
| ZNF232 | 0.720 | 0.615 | 0.357 | 96 | 65 | 56 | 36 | ZNF232 | zinc finger protein 232 | protein-coding |
| SS18L1 | 1.043 | 0.693 | 0.496 | 67 | 66 | 44 | 35 | SS18L1 | synovial sarcoma translocation gene on chromosome 18-like 1 | protein-coding |
| PIR | 0.747 | 1.086 | 2.480 | 26 | 18 | 27 | 70 | PIR | pirin (iron-binding nuclear protein) | protein-coding |
| SPECC1L-ADORA2A | 0.619 | 0.598 | 0.438 | 76 | 44 | 43 | 35 | SPECC1L-ADORA2A |  |  |
| SELPLG | 1.138 | 1.241 | 2.409 | 26 | 28 | 31 | 68 | SELPLG | selectin P ligand | protein-coding |
| TTI1 | 0.865 | 0.881 | 0.411 | 81 | 66 | 68 | 35 | TTI1 | Tel2 interacting protein 1 homolog (S. pombe) | protein-coding |
| R3HCC1L | 0.892 | 0.733 | 0.469 | 69 | 58 | 48 | 34 | R3HCC1L |  |  |
| STKLD1 | 0.983 | 0.688 | 0.468 | 69 | 64 | 45 | 34 | STKLD1 |  |  |
| B4GALT6 | 2.310 | 1.952 | 3.340 | 21 | 47 | 40 | 77 | B4GALT6 | UDP-Gal:betaGlcNAc beta 1,4- galactosyltransferase, polypeptide 6 | protein-coding |
| NOA1 | 0.853 | 0.875 | 0.476 | 66 | 53 | 55 | 33 | NOA1 |  |  |
| MIR100HG | 2.165 | 1.381 | 3.945 | 21 | 44 | 28 | 91 | MIR100HG |  |  |
| TCF7 | 0.788 | 1.084 | 0.388 | 81 | 60 | 84 | 33 | TCF7 | transcription factor 7 (T-cell specific, HMG-box) | protein-coding |
| ARHGAP32 | 1.009 | 0.542 | 0.374 | 84 | 80 | 43 | 33 | ARHGAP32 | Rho GTPase activating protein 32 | protein-coding |
| UAP1L1 | 0.653 | 0.840 | 0.370 | 85 | 52 | 68 | 33 | UAP1L1 | UDP-N-acteylglucosamine pyrophosphorylase 1-like 1 | protein-coding |
| PELI1 | 1.972 | 1.523 | 3.086 | 21 | 40 | 31 | 71 | PELI1 | pellino homolog 1 (Drosophila) | protein-coding |
| CUL9 | 0.699 | 0.746 | 0.339 | 93 | 61 | 66 | 33 | CUL9 | cullin 9 | protein-coding |
| MICAL3 | 0.888 | 0.624 | 0.321 | 98 | 82 | 58 | 33 | MICAL3 | microtubule associated monoxygenase, calponin and LIM domain containing 3 | protein-coding |
| TFF3 | 1.543 | 2.190 | 3.081 | 21 | 31 | 45 | 71 | TFF3 | trefoil factor 3 (intestinal) | protein-coding |
| ICOSLG | 0.530 | 0.743 | 0.407 | 75 | 37 | 53 | 32 | ICOSLG | inducible T-cell co-stimulator ligand | protein-coding |
| ZNF124 | 0.894 | 0.503 | 0.401 | 76 | 64 | 36 | 32 | ZNF124 | zinc finger protein 124 | protein-coding |
| TBC1D10A | 1.074 | 0.594 | 0.447 | 66 | 67 | 37 | 31 | TBC1D10A | TBC1 domain family, member 10A | protein-coding |
| ZSCAN18 | 1.078 | 0.826 | 0.275 | 108 | 110 | 85 | 31 | ZSCAN18 | zinc finger and SCAN domain containing 18 | protein-coding |
| UBR3 | 2.310 | 1.857 | 3.168 | 21 | 47 | 38 | 73 | UBR3 | ubiquitin protein ligase E3 component n-recognin 3 (putative) | protein-coding |
| ARHGAP22 | 0.597 | 0.602 | 0.334 | 86 | 48 | 49 | 30 | ARHGAP22 | Rho GTPase activating protein 22 | protein-coding |
| OFD1 | 1.086 | 0.899 | 0.360 | 77 | 79 | 66 | 29 | OFD1 | oral-facial-digital syndrome 1 | protein-coding |
| FAM229A | 0.558 | 2.888 | 3.819 | 20 | 10 | 57 | 84 | FAM229A |  |  |
| TMEM45A | 1.818 | 1.299 | 3.453 | 20 | 35 | 25 | 76 | TMEM45A | transmembrane protein 45A | protein-coding |
| FBXO31 | 1.222 | 0.549 | 0.334 | 83 | 96 | 43 | 29 | FBXO31 | F-box protein 31 | protein-coding |
| CREBRF | 1.274 | 2.929 | 4.055 | 19 | 23 | 55 | 85 | CREBRF |  |  |
| SCD5 | 0.522 | 0.988 | 0.372 | 72 | 35 | 68 | 28 | SCD5 | stearoyl-CoA desaturase 5 | protein-coding |
| ZNF587B | 0.936 | 0.576 | 0.210 | 128 | 113 | 70 | 28 | ZNF587B |  |  |
| GABPA | 2.509 | 3.194 | 3.818 | 18 | 44 | 57 | 76 | GABPA | GA binding protein transcription factor, alpha subunit 60kDa | protein-coding |
| GPR155 | 1.617 | 1.159 | 3.776 | 18 | 28 | 20 | 75 | GPR155 | G protein-coupled receptor 155 | protein-coding |
| GTF2IRD2 | 0.617 | 2.477 | 3.427 | 18 | 10 | 44 | 68 | GTF2IRD2 | GTF2I repeat domain containing 2 | protein-coding |
| MOSPD3 | 2.285 | 1.269 | 3.573 | 18 | 40 | 22 | 71 | MOSPD3 | motile sperm domain containing 3 | protein-coding |
| TADA2A | 0.997 | 0.643 | 0.374 | 69 | 65 | 42 | 27 | TADA2A | transcriptional adaptor 2A | protein-coding |
| CYP2R1 | 0.684 | 0.753 | 0.320 | 81 | 52 | 58 | 27 | CYP2R1 | cytochrome P450, family 2, subfamily R, polypeptide 1 | protein-coding |
| PLCG2 | 2.174 | 2.754 | 3.522 | 18 | 38 | 49 | 70 | PLCG2 | phospholipase C, gamma 2 (phosphatidylinositol-specific) | protein-coding |
| STX1A | 0.648 | 0.500 | 0.377 | 66 | 40 | 31 | 26 | STX1A | syntaxin 1A (brain) | protein-coding |
| ZNF227 | 1.791 | 0.582 | 0.351 | 71 | 121 | 39 | 26 | ZNF227 | zinc finger protein 227 | protein-coding |
| PRPF40B | 0.530 | 0.724 | 0.324 | 77 | 38 | 53 | 26 | PRPF40B | PRP40 pre-mRNA processing factor 40 homolog B (S. cerevisiae) | protein-coding |
| CDS1 | 1.499 | 2.833 | 4.548 | 16 | 23 | 45 | 81 | CDS1 | CDP-diacylglycerol synthase (phosphatidate cytidylyltransferase) 1 | protein-coding |
| ZNF512 | 0.774 | 0.734 | 0.363 | 66 | 48 | 46 | 25 | ZNF512 | zinc finger protein 512 | protein-coding |
| LYZ | 1.330 | 0.528 | 6.598 | 15 | 19 | 7 | 111 | LYZ | lysozyme | protein-coding |
| SDPR | 3.091 | 3.142 | 6.294 | 11 | 34 | 35 | 79 | SDPR | serum deprivation response | protein-coding |
| RND1 | 2.505 | 4.088 | 6.015 | 10 | 25 | 42 | 69 | RND1 | Rho family GTPase 1 | protein-coding |
| APOLD1 | 0.862 | 0.643 | 0.348 | 69 | 56 | 42 | 25 | APOLD1 | apolipoprotein L domain containing 1 | protein-coding |
| SNPH | 0.835 | 0.722 | 0.343 | 70 | 55 | 48 | 25 | SNPH | syntaphilin | protein-coding |
| APOE | 1.490 | 1.681 | 7.560 | 9 | 13 | 15 | 79 | APOE | apolipoprotein E | protein-coding |
| MED18 | 1.499 | 0.539 | 0.325 | 71 | 101 | 36 | 24 | MED18 | mediator complex subunit 18 | protein-coding |
| ORAI3 | 0.611 | 0.506 | 0.198 | 117 | 67 | 56 | 24 | ORAI3 | ORAI calcium release-activated calcium modulator 3 | protein-coding |
| CARD10 | 1.201 | 0.564 | 0.207 | 103 | 117 | 55 | 22 | CARD10 | caspase recruitment domain family, member 10 | protein-coding |
| ZBED1 | 0.888 | 0.505 | 0.351 | 55 | 46 | 26 | 20 | ZBED1 | zinc finger, BED-type containing 1 | protein-coding |
| IQSEC1 | 1.012 | 0.688 | 0.254 | 69 | 66 | 45 | 18 | IQSEC1 | IQ motif and Sec7 domain 1 | protein-coding |
| ZBED1 | 0.949 | 0.508 | 0.247 | 67 | 60 | 32 | 17 | ZBED1 | zinc finger, BED-type containing 1 | protein-coding |
| DHX16 | 1.000 | 1.000 | 1.000 | 0 | 0 | 0 | 0 | DHX16 | DEAH (Asp-Glu-Ala-His) box polypeptide 16 | protein-coding |
| DHX16 | 1.000 | 1.000 | 1.000 | 0 | 0 | 0 | 0 | DHX16 | DEAH (Asp-Glu-Ala-His) box polypeptide 16 | protein-coding |
| DHX16 | 1.000 | 1.000 | 1.000 | 0 | 0 | 0 | 0 | DHX16 | DEAH (Asp-Glu-Ala-His) box polypeptide 16 | protein-coding |
| DHX16 | 1.000 | 1.000 | 1.000 | 0 | 0 | 0 | 0 | DHX16 | DEAH (Asp-Glu-Ala-His) box polypeptide 16 | protein-coding |
| DHX16 | 1.000 | 1.000 | 1.000 | 0 | 0 | 0 | 0 | DHX16 | DEAH (Asp-Glu-Ala-His) box polypeptide 16 | protein-coding |
| DHX16 | 1.000 | 1.000 | 1.000 | 0 | 0 | 0 | 0 | DHX16 | DEAH (Asp-Glu-Ala-His) box polypeptide 16 | protein-coding |
| DHX16 | 1.000 | 1.000 | 1.000 | 0 | 0 | 0 | 0 | DHX16 | DEAH (Asp-Glu-Ala-His) box polypeptide 16 | protein-coding |
| FKBPL | 1.000 | 1.000 | 1.000 | 0 | 0 | 0 | 0 | FKBPL | FK506 binding protein like | protein-coding |
| MAFB | 1.829 | 7.779 | 15.268 | 6 | 11 | 51 | 112 | MAFB | v-maf musculoaponeurotic fibrosarcoma oncogene homolog B (avian) | protein-coding |
| FKBPL | 1.000 | 1.000 | 1.000 | 0 | 0 | 0 | 0 | FKBPL | FK506 binding protein like | protein-coding |
| FKBPL | 1.000 | 1.000 | 1.000 | 0 | 0 | 0 | 0 | FKBPL | FK506 binding protein like | protein-coding |
|  |  |  |  |  |  |  |  |  |  |  |

Supplementary Table 2. Top 5 Ingenuity Cannonical Pathway

| **Treatment** | **Ingenuity Canonical Pathways** | **-log(p-value)** | **Ratio** | **Molecules** |
| --- | --- | --- | --- | --- |
| **ATZ-502** | Estrogen Receptor Signaling | 2.950 | 0.039 | PELP1,NRAS,TRRAP,NCOA1,G6PC3 |
|  | Osteoarthritis Pathway | 2.720 | 0.028 | EPAS1,SPHK1,VEGFC,CASP8,NOTCH1,FZD7 |
|  | Bladder Cancer Signaling | 2.690 | 0.046 | NRAS,MMP14,VEGFC,FGF1 |
|  | Unfolded protein response | 2.340 | 0.055 | HSP90B1,HSPA5,EIF2A |
|  | HIF1α Signaling | 2.230 | 0.034 | NRAS,MMP14,NCOA1,VEGFC |
| **Doxorubicin** | Prolactin Signaling | 3.455 | 0.004 | CEBPB, CREBBP, IRF1, MAP2K1, NR3C1, SOCS4, SOCS7 |
|  | Role of IL-17F in Allergic Inflammatory Airway Diseases | 3.150 | 0.007 | CERB1, CREBBP, CXCL1, MAP2K1, RELA |
|  | Sumoylation Pathway | 3.073 | 0.008 | CERBBP, NR3C1, RCOR1, RHOG, RND3, RND4, ZNF217 |
|  | Role of Macrophages, Fibroblasts and Endothelial Cells in Rheumatoid Arthritis | 2.816 | 0.015 | CEBPB, CEBPG, CERB1, CREBBP, FZD3, IL1RAP, LTBR, MAP2K1, PRSS3, RELA, RIPK1, TRAF4, VEGFA |
|  | Osteoarthritis Pathway | 2.699 | 0.021 | ANXA5, CEBPB, CREB1, CREBBP, FZD3, IL1RAP, PTCH1, RELA, SOX9, VEGFA, |
| **Combination** | Inositol Pyrophophates Biosynthesis | 3.761 | 0.002 | IP6K1, IPMK, IPPK |
|  | 1D-myo-inositol Hexakisphophate Biosythesis V | 3.182 | 0.007 | IPMK, IPPK |
|  | Dermatan Sulfate Biosythesis | 2.734 | 0.019 | CHST2, CHST15, DSEL, NDST2, XYLT2 |
|  | Notch Siganling | 2.621 | 0.024 | HES1, MAML1, NCSTN, NUMBL |
|  | Dermatan Sulfate Biosynthesis | 2.317 | 0.048 | CHST2, CHST15, DSEL, NDST2 |
| The ratio is calculated as follows: # of genes in a given pathway that meet your cutoff criteria, divided by the total # of genes that make up that pathway and that are in the reference gene set. | | | | |

Supplementary Table 3. Combination Index (CI) values for doxorubicin and ATZ-502 combination with various cancer cell lines.

| **Cell line** | **Concentration (uM)** | | **Combination  effect** | **CI value** |
| --- | --- | --- | --- | --- |
|  | **Doxorubicin** | **ATZ-502** |  |  |
| **MDA-MB 231** | 10.0 | 0.5 | 0.09 | 0.08988 |
|  | 2.0 | 0.5 | 0.32 | 0.29357 |
|  | 0.4 | 0.5 | 0.42 | 0.12703 |
|  | 0.08 | 0.5 | 0.65 | 0.13723 |
|  | 0.016 | 0.5 | 0.95 | 1.80054 |
| **SiHa** | 10.0 | 0.5 | 0.06 | 0.17746 |
|  | 2.0 | 0.5 | 0.25 | 0.59413 |
|  | 0.4 | 0.5 | 0.35 | 0.3721 |
|  | 0.08 | 0.5 | 0.62 | 0.61517 |
|  | 0.016 | 0.5 | 0.89 | 1.86364 |
| **SK-OV 3** | 10.0 | 0.01 | 0.02 | Infinit |
|  | 2.0 | 0.01 | 0.08 | Infinit |
|  | 0.4 | 0.01 | 0.47 | Infinit |
|  | 0.08 | 0.01 | 0.68 | NaN |
|  | 0.016 | 0.01 | 0.87 | NaN |
| **PC3** | 10.0 | 0.01 | 0.001 | Infinit |
|  | 2.0 | 0.01 | 0.05 | Infinit |
|  | 0.4 | 0.01 | 0.43 | Infinit |
|  | 0.08 | 0.01 | 0.58 | NaN |
|  | 0.016 | 0.01 | 0.93 | NaN |
| **MCF-7/ADR** | 10.0 | 0.5 | 0.36 | 0.19281 |
|  | 2.0 | 0.5 | 0.53 | 0.19281 |
|  | 0.4 | 0.5 | 0.53 | 0.15048 |
|  | 0.08 | 0.5 | 0.61 | 0.18384 |
|  | 0.016 | 0.5 | 0.99 | 5.23978 |
