# Supplementary figures and images for: Inhibition of NUPR1–Karyopherin β1 Binding Increases Anticancer Drug Sensitivity
Source: Int J Mol Sci. 2021 Mar 10;22(6):2794. doi: 10.3390/ijms22062794 (PMC8000408; doi:10.3390/ijms22062794)

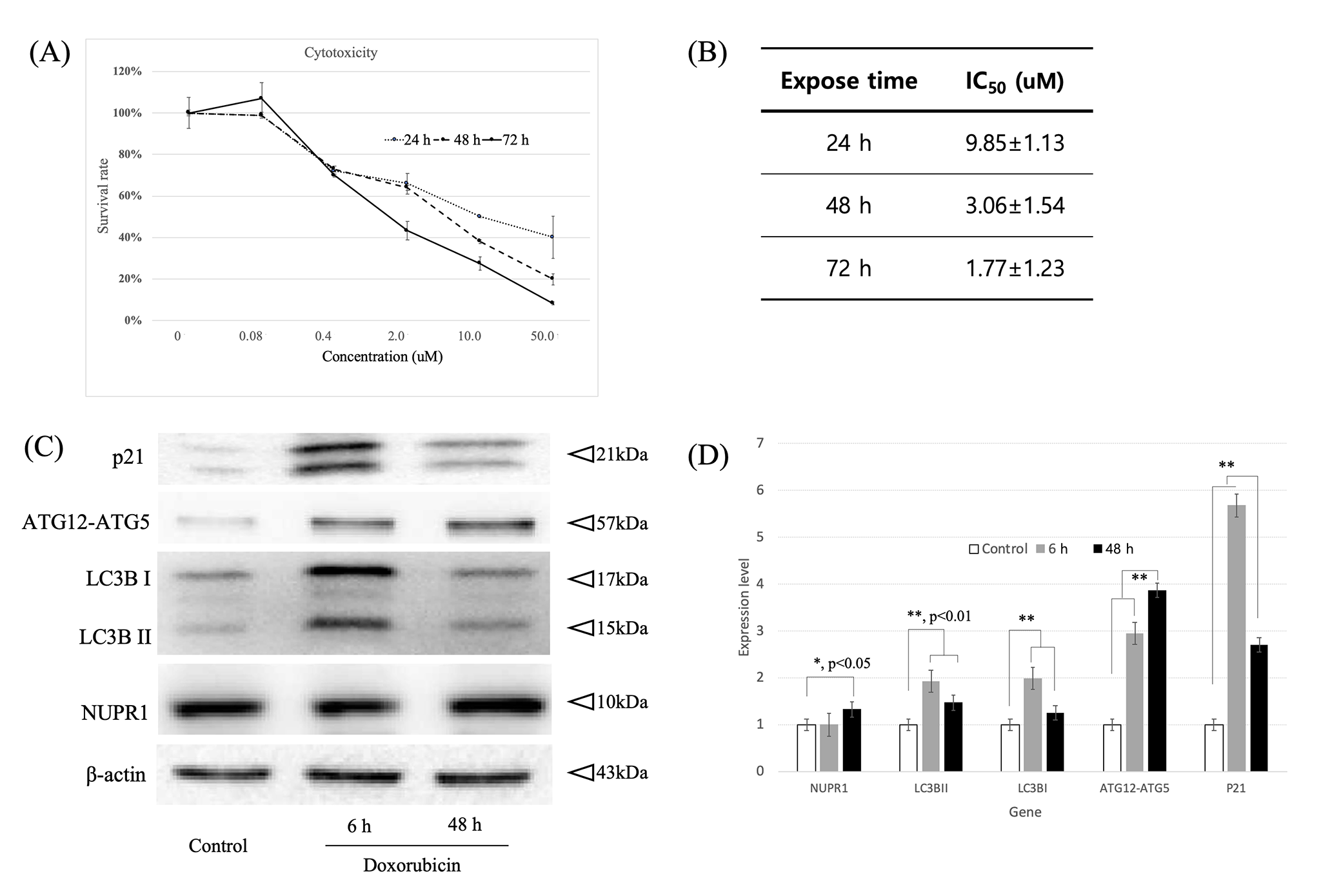

Supplement: Supplementary file 1 [file ijms-22-02794-s001.zip › Supplementary Materials /Figures/Figure 1.tif]

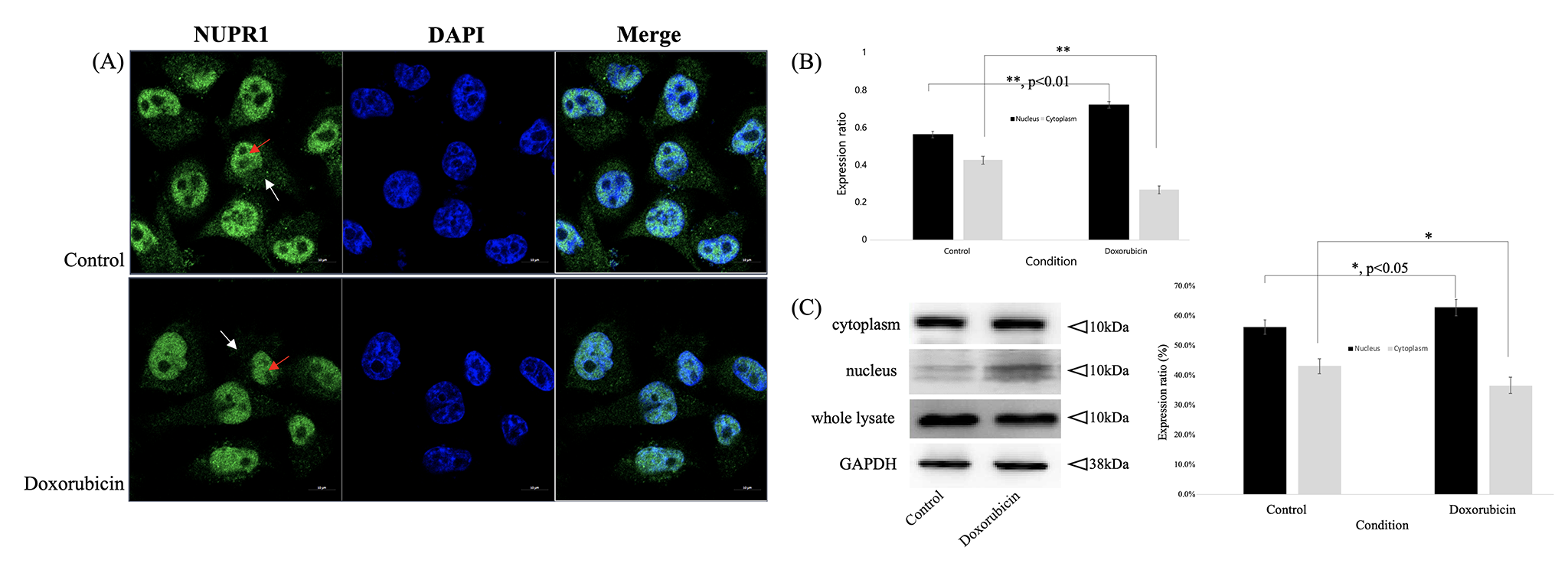

Supplement: Supplementary file 1 [file ijms-22-02794-s001.zip › Supplementary Materials /Figures/Figure 2.tif]

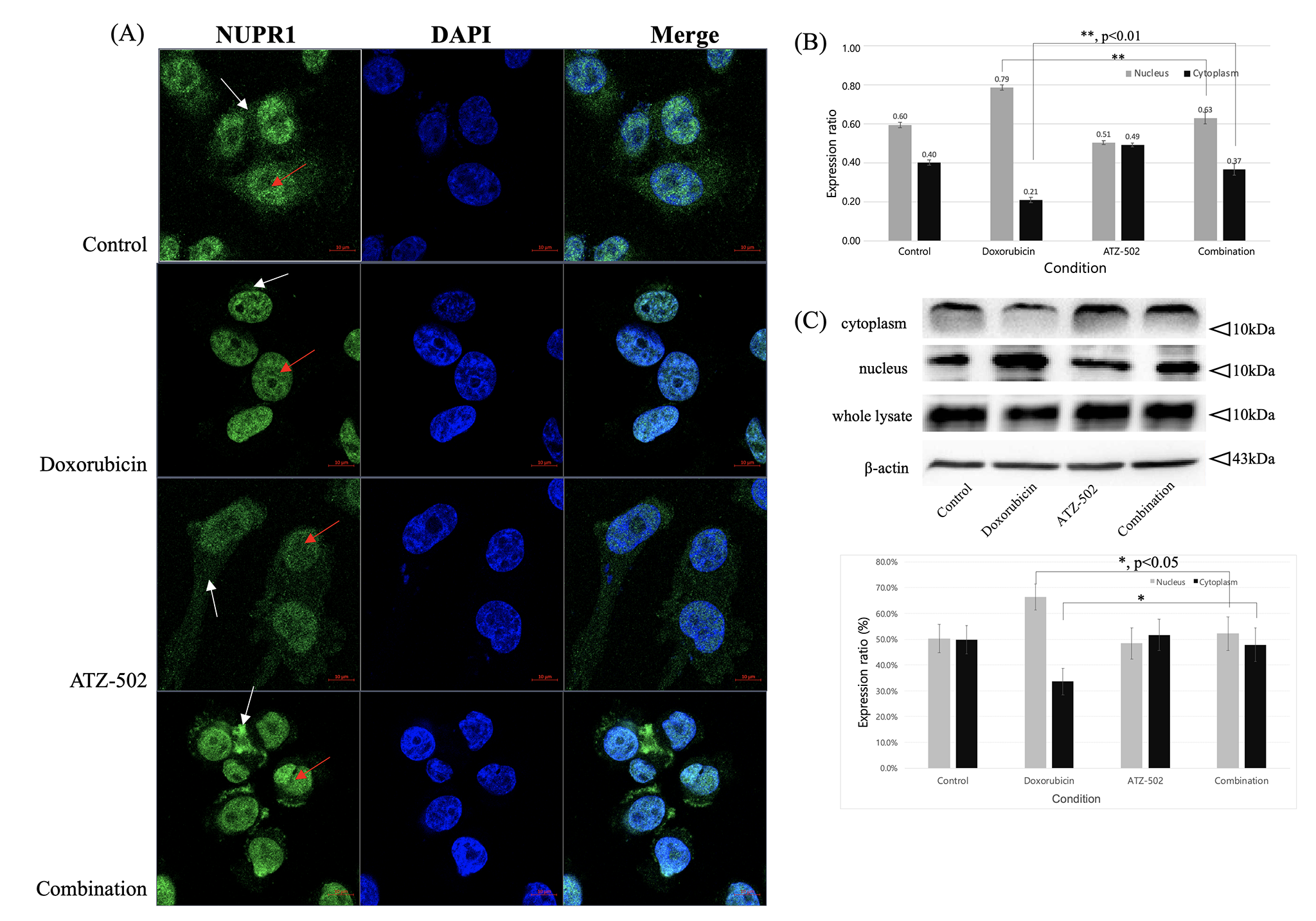

Supplement: Supplementary file 1 [file ijms-22-02794-s001.zip › Supplementary Materials /Figures/Figure 3.tif]

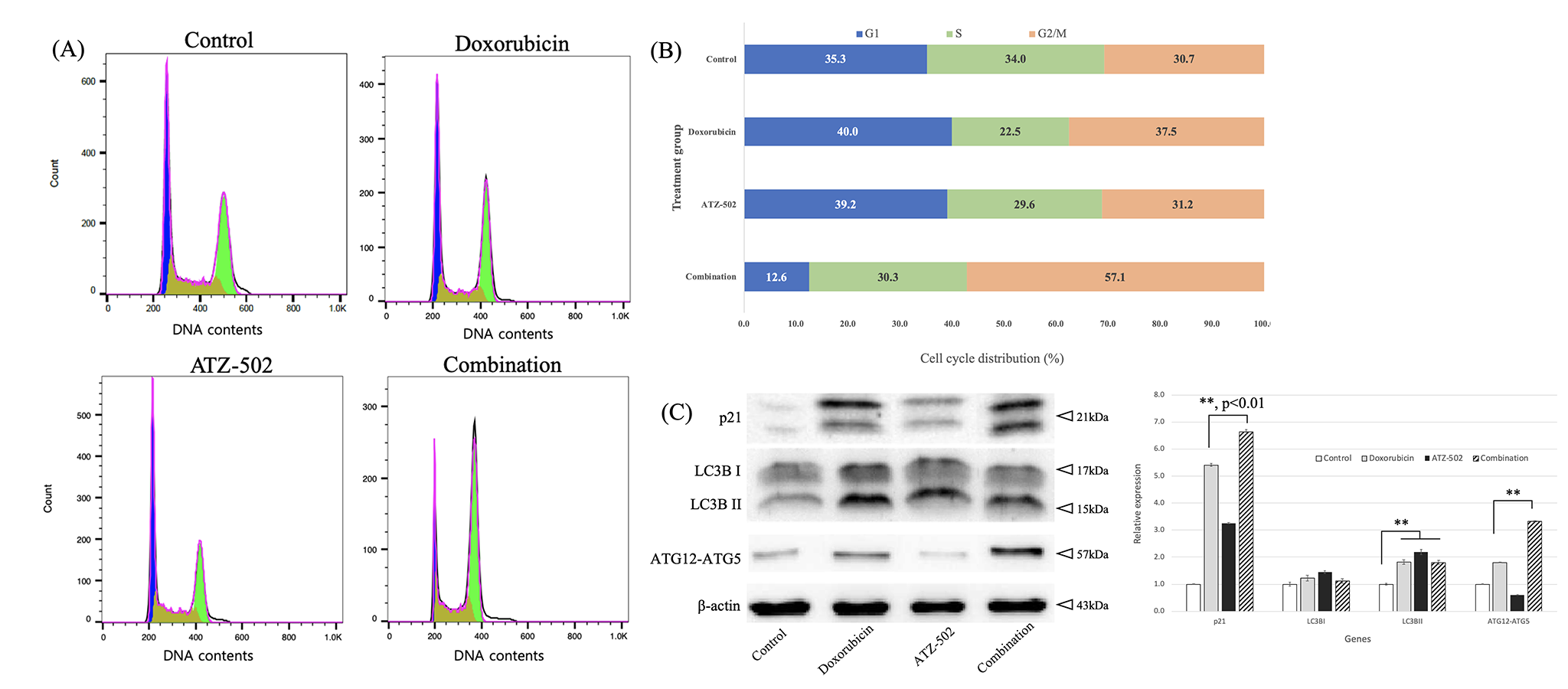

Supplement: Supplementary file 1 [file ijms-22-02794-s001.zip › Supplementary Materials /Figures/Figure 4.tif]

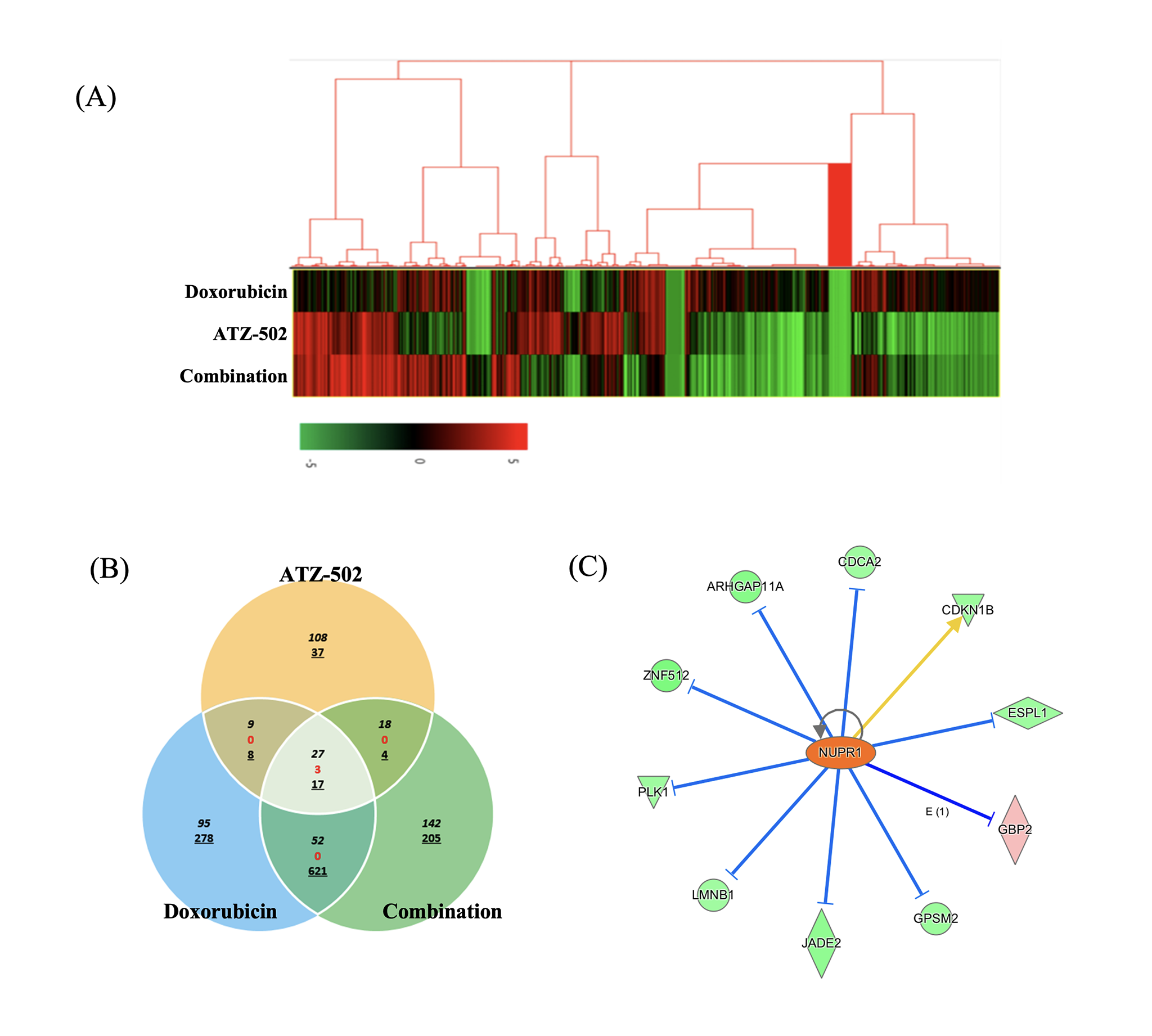

Supplement: Supplementary file 1 [file ijms-22-02794-s001.zip › Supplementary Materials /Figures/Figure 5.tif]

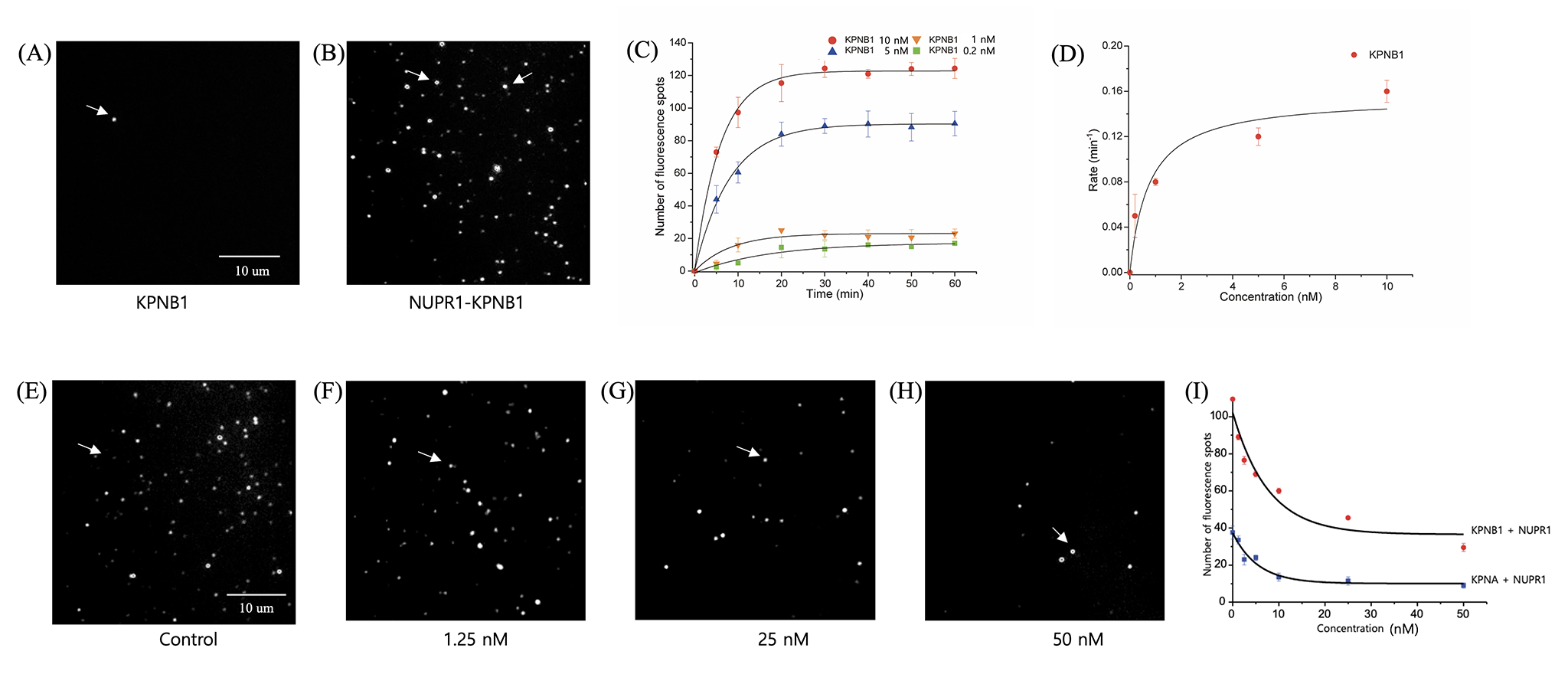

Supplement: Supplementary file 1 [file ijms-22-02794-s001.zip › Supplementary Materials /Figures/Figure 6.tif]
